# *APOE* ε4, physical activity, and the brain: a review of systematic reviews

**DOI:** 10.3389/fnagi.2026.1798639

**Published:** 2026-07-23

**Authors:** Nada Ali, Noelle Chakbazof, Sara Ghasem Pour, Lorena Contreras, Jimena Estrada, Gene E. Alexander, David A. Raichlen, Hussein N. Yassine

**Affiliations:** 1 Department of Neurology, University of Southern California, Los Angeles, CA, United States; 2 Department of Psychology, University of Arizona, Tucson, AZ, United States; 3 Department of Psychiatry, University of Arizona, Tucson, AZ, United States; 4 Neuroscience Graduate Interdisciplinary Program, University of Arizona, Tucson, AZ, United States; 5 Physiological Sciences Graduate Interdisciplinary Program, University of Arizona, Tucson, AZ, United States; 6 Evelyn F. McKnight Brain Institute, University of Arizona, Tucson, AZ, United States; 7 Arizona Alzheimer’s Consortium, Phoenix, AZ, United States; 8 Department of Biological Sciences, University of Southern California, Los Angeles, CA, United States

**Keywords:** Alzheimer’s disease, APOE4 allele, exercise, physical activity, vascular

## Abstract

**Background:**

Physical activity (PA) is often proposed as a modifiable strategy to reduce cognitive decline and dementia risk, particularly among individuals at elevated genetic risk for Alzheimer’s disease (AD). However, it remains unclear whether the existing literature tests PA at the disease stage and in the populations most likely to show benefit.

**Objective:**

This umbrella review evaluated whether associations between PA and cognitive, fluid biomarker, neuroimaging, and vascular/metabolic outcomes differ by apolipoprotein E ε4 (*APOE* ε4) genotype across stages of cognitive aging, with particular attention to how study design and baseline cognitive status shape interpretation of the evidence.

**Methods:**

Systematic reviews and meta-analyses were screened for primary studies examining PA in adults classified by baseline cognitive status as cognitively unimpaired, mild cognitive impairment (MCI), or dementia. Primary studies reporting *APOE* ε4-stratified outcomes were extracted and qualitatively synthesized by outcome domain, cognitive stage, and study design.

**Results:**

Of 2,100 records identified, seven systematic reviews met inclusion criteria, yielding 68 unique primary studies. Favorable associations between PA and cognitive, biomarker, neuroimaging, and vascular/metabolic outcomes were most often reported in observational studies of younger or cognitively unimpaired adults. Several studies suggested stronger associations among *APOE* ε4 carriers, including midlife cognitive associations, neuroimaging markers, and vascular/metabolic outcomes such as lipid profiles. In contrast, randomized controlled trials were few, generally enrolled older adults with MCI or dementia, included small *APOE* ε4 subgroups, and reported largely null or mixed genotype-specific effects.

**Conclusion:**

The current evidence does not establish a definitive *APOE* ε4-specific preventive effect of PA. Future studies may be most informative if they target earlier-stage, low-active, or metabolically at-risk *APOE* ε4 carriers using objective PA measures and proximal vascular, metabolic, imaging, or blood-based biomarker outcomes.

## Introduction

Physical inactivity has been estimated, based largely on epidemiological models, to contribute to approximately 13% of Alzheimer’s disease (AD) cases worldwide (Barnes and Yaffe, 2011). However, the precise contribution of physical activity (PA) to dementia prevention or treatment remains elusive due to inconsistent research findings. Observational studies suggest that PA is associated with reduced AD risk and markers of neurometabolic health, including greater hippocampal regional volumes, lower neuro-inflammatory markers, and more favorable lipid metabolism regulation (Santos-Lozano et al., 2016; Domingos et al., 2021). Evidence from randomized controlled trials (RCTs) assessing the relationship between PA and cognitive function shows predominantly null results (Eggermont et al., 2009; Espeland et al., 2017; Karssemeijer et al., 2019). This discrepancy makes it difficult to determine whether PA has a true disease-modifying role, whether its benefits depend on the disease stage at which it is introduced, or whether the current evidence base is not well aligned with the populations and outcomes most likely to show response.

Presence of the apolipoprotein ε4 (*APOE* ε4) allele, the strongest genetic risk factor for late-onset sporadic AD, may modulate the effect of PA on cognitive function and brain structure. Although 40% of AD cases consist of *APOE* ε4 carriers, there is a lack of personalized lifestyle interventions for this high-risk group (Farrer et al., 1997; Livingston et al., 2020). Previous literature has been conflicting, with several studies reporting potential protective associations of PA with dementia risk in *APOE* ε4 carriers, while others report such benefits among non-carriers only (De Frutos-Lucas et al., 2020b). These inconsistencies may reflect differences in study design, baseline cognitive status, age, PA measurement, intervention timing, and outcome selection rather than a single uniform effect of *APOE* ε4. Yet, differences between genetic *APOE* status and regional brain activation in response to PA have also been described, with *APOE* ε4 carriers exhibiting greater activation in memory-related brain regions, such as the hippocampus and posterior cingulate cortex (Smith et al., 2011). Further, PA has been proposed as a possible modulatory factor on AD pathology (e.g., neuroinflammation, lipid metabolism, neurotrophic factors) in *APOE* ε4 carriers, with potential to modify AD-related pathological processes, (Tokgöz and Claassen, 2021). These findings suggest that PA may be most informative to study earlier in the disease continuum, before substantial neurodegeneration has occurred.

Several systematic reviews have evaluated the association between PA, cognition, and *APOE* ε4 status; however, no umbrella review has synthesized *APOE*-specific primary studies while explicitly accounting for baseline cognitive status. To address this gap, the present umbrella review examines primary studies extracted from systematic reviews to evaluate whether cognitive, fluid biomarker, neuroimaging, and vascular/metabolic associations and effects of PA differ by *APOE* ε4 genotype across stages of cognitive aging. We focus on whether apparent *APOE* ε4-specific findings vary by baseline cognitive status and study design, and whether the current evidence is aligned with the disease stage at which PA may be most informative.

## Methods

For the purposes of this review, outcomes are defined to include cognitive function and neuropsychological performance; fluid biomarkers, including blood- and CSF-based measures; neuroimaging markers of brain structure, function, and pathology; and vascular/metabolic markers such as lipid profiles and blood pressure-related measures. This terminology encompasses what has been variably described in primary studies as “neurocognitive,” “neuro-cognitive,” or “cognitive” outcomes, and is used consistently throughout this review to reflect the full scope of measures examined.

### Protocol and registration

The protocol of the following review was registered in the Open Science Framework (https://doi.org/10.17605/OSF.IO/T56SC) before analyses were performed. Ethics approval was not necessary as the review did not include analysis of identifiable data.

### Literature search

The search strategy was developed using Medical Subject Headings (MeSH) terms with help from USC medical librarians and included the following databases: PubMed/MEDLINE (coverage: 2000–2023), Embase (coverage: 2000–2023), Web of Science (coverage: 2000–2023), and Scopus (coverage: 2000–2023) to generate relevant titles and abstracts. A detailed search strategy is provided in the Supplementary Appendix. All citations were imported into Covidence^1^ for removal of duplicates and further screening.

### Search strategies for inclusion

This review was designed to identify systematic reviews and meta-analyses on the effects of PA and exercise interventions, as well as observational PA exposure, in adults with unimpaired cognition, MCI, or dementia. Moreover, the following search strategy sought to identify reviews that studied the association or effect of PA (e.g., aerobic exercise) and *APOE* ε4 on cognitive, fluid biomarker, neuroimaging, and vascular/metabolic outcomes.

We used a structured search strategy guided by the PICO framework. Inclusion criteria at the abstract level were that the study: (1) was a systematic review or meta-analysis; (2) was published in English; (3) included a study involving any form of PA exposure or exercise intervention; and (4) reported outcomes related to cognition, fluid biomarkers, neuroimaging markers, vascular/metabolic markers, or the *APOE* ε4 allele. For full-text screening, additional criteria included: (1) publication year 2000 or later; (2) focus on adult populations of any gender, with at least one group consisting of *APOE* ε4 carriers or individuals at high risk for dementia; (3) PA exposure or intervention involving aerobic or cardiorespiratory exercise; and (4) comparison group, exposure category, or control condition including either no exercise, lower PA, or anaerobic/strength training or flexibility-based PA. A detailed list of search terms is provided in the Supplementary Appendix.

### Data extraction and categorization

From each of the included systematic reviews, primary study data, including study design, participant characteristics (e.g., age, cognitive status at baseline, number of *APOE* ε4 carriers), PA or exercise methods, and cognitive, fluid biomarker, neuroimaging, and vascular/metabolic outcomes were extracted (Supplementary Table 1). Primary studies were categorized in accordance with participant baseline cognitive status and outcome domain.

### Data synthesis

Findings were synthesized qualitatively using a narrative approach, with primary studies grouped by baseline cognitive status (cognitively unimpaired, MCI, dementia, or mixed cognitive status), outcome domain (cognition, fluid biomarkers, neuroimaging markers, and vascular/metabolic markers), study design, and *APOE* ε4 carrier status.

### Assessment of overlap

Overlapping studies were identified and duplicate searches were removed.

### Methodological quality

A quality appraisal of extracted systematic reviews was performed using the Measurement Tool to Assess Systematic Reviews (AMSTAR) 2.

### Risk of Bias

Two independent reviewers (AR, SS) conducted the AMSTAR 2 review to reduce bias. A third independent reviewer (LC) was consulted to achieve a consensus on disagreements.

## Results

**Figure 1:**
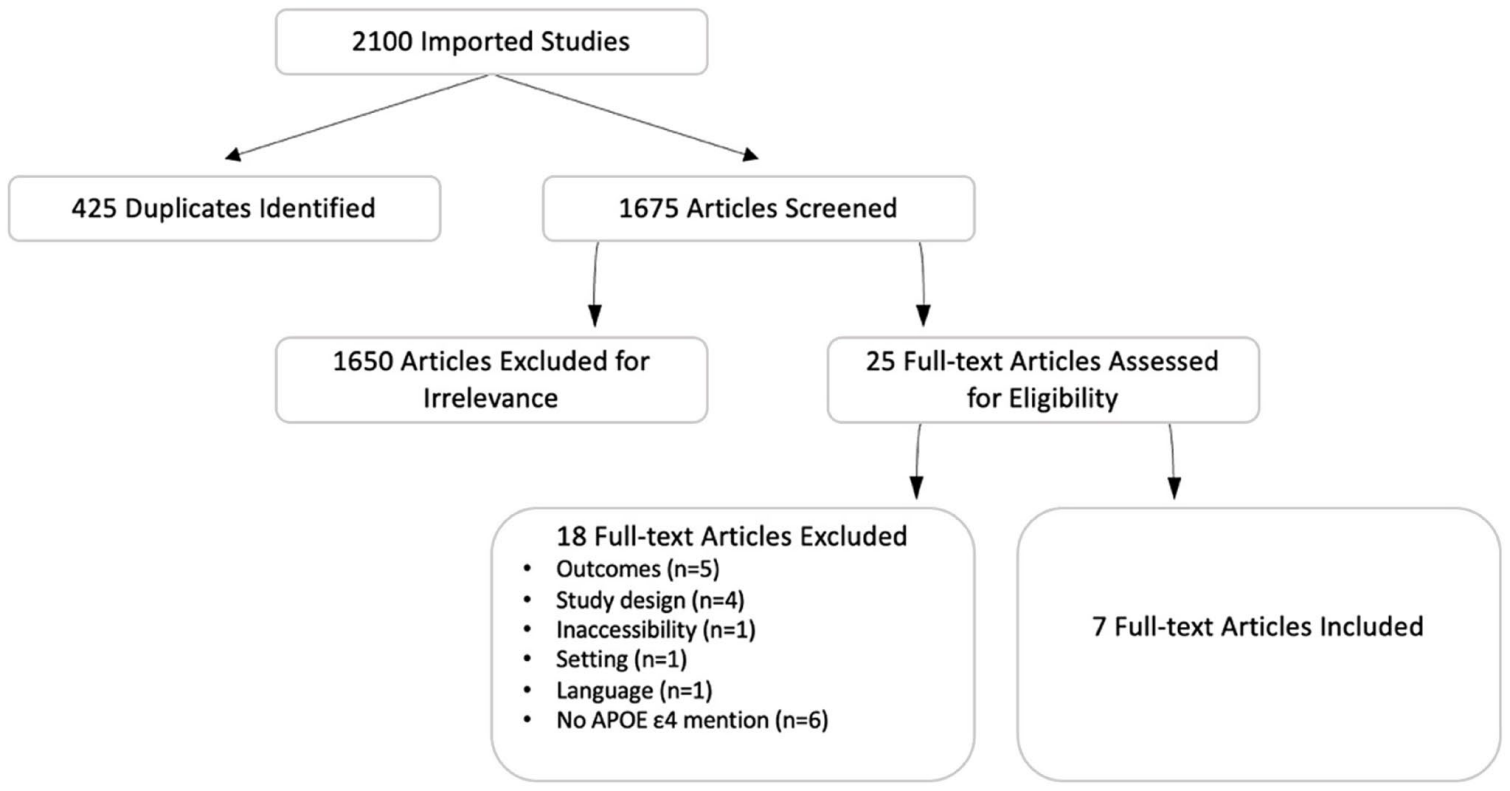
Preferred reporting items for systematic reviews and meta-analyses (PRISMA) flow chart of the study selection and inclusion process. A sum of 2,100 studies was imported for initial screening. A total of 1,675 studies remained for title and abstract screening following removal of duplicates, of which, 1,650 did not meet the eligibility criteria. The remaining 25 full-text articles were reviewed; seven studies were ultimately included in the analysis.

**Figure 2:**
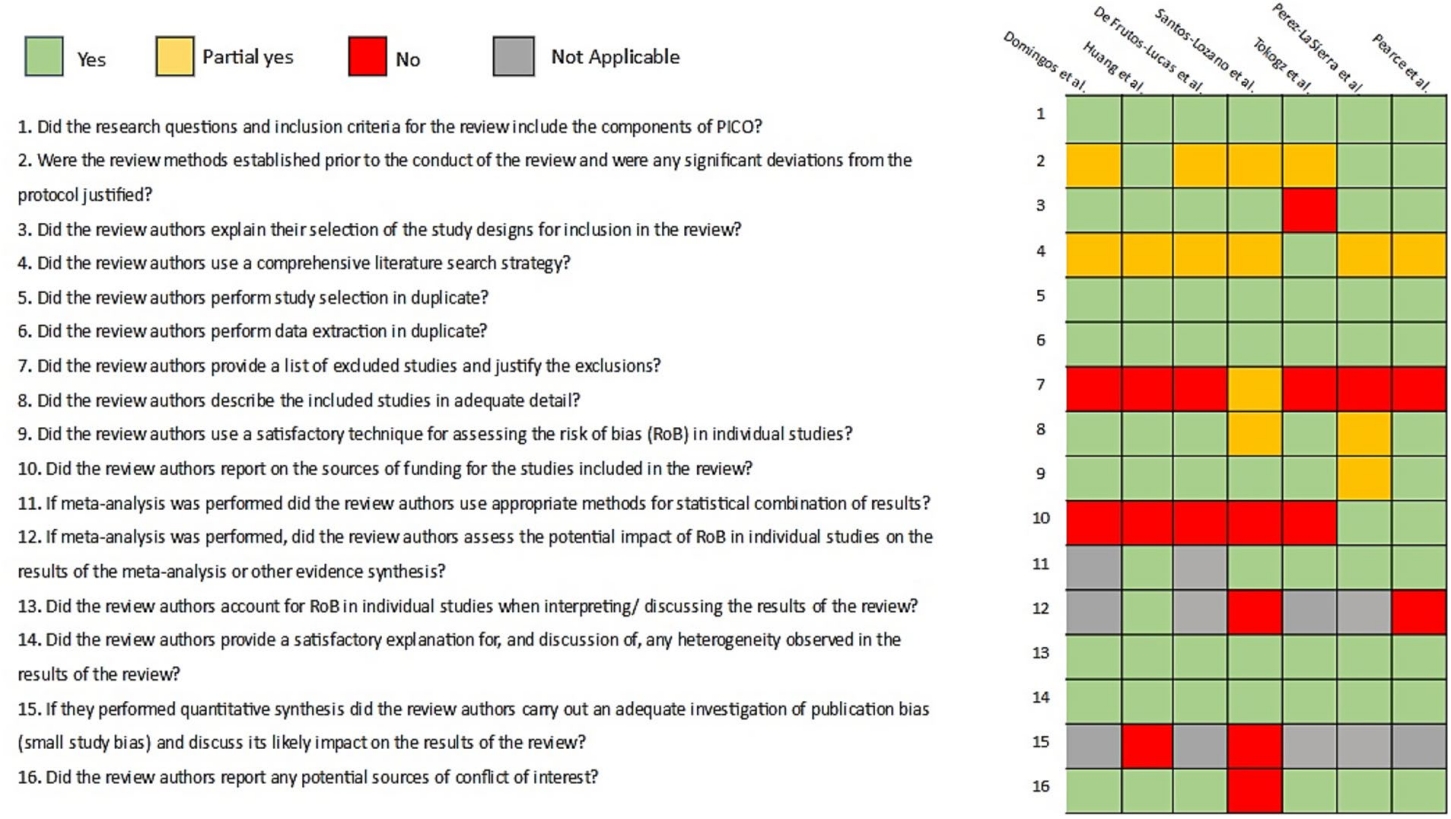
Detailed overview of AMSTAR 2 assessment for each of the 7 extracted articles.

This review was conducted in adherence with Preferred Reporting Items for Systematic Reviews and Meta-Analyses (PRISMA) guidelines for study selection. A total of 2,100 studies was imported for initial screening. A total of 1,675 studies remained for title and abstract screening following removal of duplicates, of which, 1,650 did not meet the eligibility criteria. The remaining 25 full-text articles were reviewed; seven studies were ultimately included in the analysis. Full text articles were excluded due to: (a) irrelevant outcomes (*n* = 5), (b) unsuitable study design (*n* = 4), (c) inaccessibility (*n* = 1), (d) ineligible study setting (*n* = 1), (e) incompatible language (*n* = 1), and (f) did not account for *APOE* ε4 genotype (*n* = 6) (Figure 1). Appraisal of the 7 reviews was conducted using AMSTAR2 criteria (Figure 2).

### Characteristics of primary studies included across systematic reviews

A sum of 170 primary studies was extracted across all included systematic reviews. Following deduplication, the total number of individual studies was 78. Ten studies were further excluded as they either did not specify *APOE* results or the number of *APOE* ε4 carriers, leaving a remainder of 68 primary studies for review. Of the primary studies eligible for review, PA was largely assessed using self-report measures, while a limited number used objective measures such as accelerometry. The following review highlights findings on the relationship between PA, cognitive and brain health, and *APOE* status.

#### Primary studies of cognitively unimpaired adults

##### Cognition outcome(s)

Primary studies across systematic reviews with a cognitively unimpaired sample at baseline (*N* = 39) were all observational studies except for one RCT. Of observational studies focusing on cognitive function as a main outcome (*N* = 21), most reported a modifying effect of *APOE* genotype on the association of PA with cognitive outcomes (*N* = 13). Beneficial effects of PA on cognitive outcomes of MCI and dementia risk, for example, were observed among *APOE* ε4 carriers more often (*N* = 8) (Krell-Roesch et al., 2016; Rovio et al., 2005; Shaaban et al., 2019; Shih et al., 2018; Ferrari et al., 2013; Luck et al., 2014; Etnier et al., 2007; Niti et al., 2008) than in non-carriers alone (*N* = 5) (Fenesi et al., 2017; Podewils et al., 2005; Tan et al., 2017; Gelber et al., 2012; Chang et al., 2010).

Observational studies indicated a pronounced protective association between midlife PA and cognitive function in cognitively unimpaired *APOE* ε4 carriers (Krell-Roesch et al., 2016; Shih et al., 2018). Conversely, primary studies of PA with mixed or unspecified cognitive samples at baseline yielded conflicting results, with some studies observing PA-cognitive associations only in *APOE* ε4 carriers (hetero- and homozygotes) (Niti et al., 2008; Kivipelto et al., 2008; Schuit et al., 2001), and other studies observing significant effects for only non ε4**-**carriers (Gelber et al., 2012; Tolppanen et al., 2015; Yang et al., 2015).

Among studies reporting on the association of PA on cognitive outcomes in cognitively unimpaired persons by *APOE* status, the largest was a 26-year longitudinal AGES—Reykjavik cohort study involving 4,945 participants (ε4+, *N* = 583) with an average age of 51 years at midlife examination and 76 years at the current examination (Chang et al., 2010). Findings showed that regular midlife PA was linked to better cognitive performance and a reduced risk of dementia at follow-up among non-ε4 carriers, whereas ε4 carriers who reported midlife PA did not exhibit a reduced risk of dementia compared to ε4 carriers who did not report midlife PA. Another observational study with an average sample age range of 65–79 years at re-examination (*N* = 1,449), which included 438 *APOE* ε4 carriers, reported a significant association between leisure-time PA (≥2x/week) and reduced dementia risk, with more pronounced benefits for ε4 carriers compared to non-carriers (Rovio et al., 2005).

Of note, several primary studies of cognitively unimpaired adults assessed associations with cognition outcomes controlling for a number of modifiable risk factors for dementia (e.g., vascular health factors) as main outcomes of interest. One cohort study of 1,438 participants with a mean age of 70.0 (6.6) years (ε4+, *N* = 201) found that *APOE* ε4 carriers with low PA had a threefold risk of dementia/cognitive impairment not dementia (CIND) versus non-carriers with high PA. Low PA *APOE* ε4 carriers with diabetes had a nearly tenfold elevated risk of dementia or cognitive impairment compared to active non-carriers who were not diabetic (Shih et al., 2018). Another prospective cohort study with a mean age of 80.4 (4.56) years controlled for diabetes as a vascular risk factor along with hypertension, among others, and found an additive interaction for high education, absence of vascular factors, and high leisure activity with *APOE* ε4 on dementia risk. Specifically, authors reported that ε4 carriers with high education, no vascular risk factors, or high leisure activities had a delay in dementia onset (1.2 years) compared to carriers with vascular factors, low education, or low leisure activity, providing a similar dementia-free survival time comparable to that of non-carriers (Ferrari et al., 2013).

A number of primary studies with cognitively unimpaired samples at baseline did not report differences of the PA association with cognitive outcomes by *APOE* status (N = 7), although several of these studies reported associations between high self-reported PA and reduced dementia or mortality risk (Taaffe et al., 2008; Hansson et al., 2019; Scarmeas et al., 2009; Scarmeas et al., 2011). Authors attributed the lack of a moderating effect of *APOE* genotype on the PA-cognition association to differences in sample composition (e.g., ethnicity, sex differences), though the inclusion of older cohorts, as most included average sample ages above 75 years, may have also contributed to the null findings.

##### Fluid/neuroimaging biomarker outcome(s)

Twenty-seven studies with a cognitively unimpaired sample at baseline incorporated fluid biomarkers, neuroimaging biomarkers, and/or vascular/metabolic markers as a main outcome of interest. Peripheral fluid biomarkers examined across these studies included plasma amyloid-beta (Aβ), plasma antioxidant capability (AOC), erythrocyte membrane fluidity, phosphatidylcholine (PC), and lipase activities (HLA, LPLA). Vascular/metabolic markers included lipid profiles (LDL-C, HDL-C, TG, TC) and systolic blood pressure. Central CSF biomarkers included CSF Aβ42. Peripheral measures and vascular/metabolic markers may reflect vascular and metabolic pathways but may not directly reflect AD pathology. Neuroimaging outcomes included Pittsburgh compound B (PiB) PET binding, PET amyloid brain deposition, and cerebral glucose uptake with positron emission tomography (PET), as well as semantic memory activation, functional connectivity, hippocampal volume and integrity, and cerebral blood flow measured with functional and structural MRI.

Studies with a cognitively unimpaired sample at baseline incorporated AD and AD-related biomarkers as a main outcome of interest (*N* = 19), three of which observed *APOE* status as a potential modifier of the association between PA and AD-related markers of amyloid-beta (Aβ) or individual alpha peak frequency (IAPF) (Brown et al., 2013; De Frutos-Lucas et al., 2018; Liang et al., 2010). These studies included older samples aged 60–95 years and associations were primarily observed among non-carriers, in which higher PA levels were associated with lower plasma Aβ [sample mean age: 69.6 (6.8)] (Brown et al., 2013), and (IAPF), a neurophysiological marker measured with magnetoencephalography (MEG) that is linked to improved brain function [sample mean age: 71.84(4.28)] (De Frutos-Lucas et al., 2018). Another study with a sample between 55 and 88 years reported that participants who met American Heart Association exercise guidelines (30 min of moderate exercise 5 days/wk) showed lower Pib PET binding [mean age: 70.39 (10)] and higher CSF Aβ42 levels [mean age: 66.46 (8.70)], with more pronounced associations in *APOE* ε4 non-carriers, although the difference between *APOE* ε4 carriers and non-carriers was not significant (Liang et al., 2010). Another study inclusive of a younger sample overall (45–88 years) reported that sedentary lifestyle (<30 min of moderate exercise 5 days/wk) was associated with increased PiB PET binding [mean age: 66.53 (9.79)] for *APOE* carriers, with a significant genotype by exercise engagement interaction for cerebral amyloid burden (Head et al., 2012). The remainder of the studies found no significant moderating effect of *APOE* status on PA and related biomarker outcomes (Stojanovic et al., 2020; Boer et al., 1997; Tsai et al., 2021; Tsai et al., 2019; Corlier et al., 2018).

Studies predominantly focusing on outcomes of lipid metabolism among younger adult samples (*N* = 8) (Bernstein et al., 2002; Boer et al., 1998; Corella et al., 2001; Gustavsson et al., 2012; Lee et al., 2018; Pisciotta et al., 2003; Schmitz et al., 2001; St-Amand et al., 1999) largely reported more protective effects of PA on lipid profiles specific to male *APOE* ε4 carriers (Corella et al., 2001) and carriers irrespective of sex (Bernstein et al., 2002; Boer et al., 1998; Lee et al., 2018; Pisciotta et al., 2003). These studies primarily included middle- to older-aged adult participants below the age of 75 years. Vascular benefits, such as improvements in low-density lipoprotein cholesterol (LDL-C), high-density lipoprotein cholesterol (HDL-C), and/or triglyceride (TG) levels were observed among *APOE* ε4 carriers with high PA engagement (Bernstein et al., 2002; Boer et al., 1998; Corella et al., 2001; Pisciotta et al., 2003). Of note, in a 6-month exercise training study, systolic blood pressure (SBP) decreased in *APOE* ε4 carriers (*N* = 15) among a sample of 28 patients with a history of stroke [mean age: 56.73 (9.71)]. Total cholesterol (TC), LDL-C, and TG levels decreased, and HDL-C increased in both genotype groups, with greater reductions of TC, LDL-C, and TG in *APOE* ε4 carriers compared to non-carriers (Lee et al., 2018). Conversely, one study with participants aged 25–48 years [female mean age: 35.25 (4.58); male mean age: 36.54 (3.25)] reported favorable lipid profiles associated with improved fitness as measured by V̇O_2max_ for *APOE* ε4 non-carriers (St-Amand et al., 1999). The remaining studies herein did not report interactions between PA level and *APOE* status on lipid levels (Gustavsson et al., 2012; Schmitz et al., 2001).

One primary study reported effects of 6-month aerobic exercise training on lipase activities in participants between 18 and 70 years [mean age: 36.77 (11.07)], with overall reductions in hepatic lipase activities (HLA) across all *APOE* genotypes. After accounting for insulin as a covariate, increased lipoprotein lipase activities (LPLA) was observed in *APOE* ε2/ε3 and ε4/ε3 carriers, while ε3 homozygotes displayed decreased levels (Seip et al., 1985). Another study which included participants between the ages of 20–70 years reported that physically active individuals displayed higher plasma AOC than non-active participants among *APOE* ε4 carriers only, with effects on erythrocyte membrane fluidity only significant in non-carriers. PC levels were not associated with PA (Piccarducci et al., 2019).

Imaging outcomes were analyzed in a number of studies (*N* = 13), many of which (*N* = 10) found an association between neuroimaging, *APOE* status, and PA. Notably, in *APOE* ε4 carriers, higher levels of PA were associated with lower PET amyloid brain deposition [mean age: 69.6(6.8)]; greater sedentary time was associated with increased deposition [mean age: 66.53 (9.79)]; these associations were not observed in non-carriers (Brown et al., 2013; Head et al., 2012). In *APOE* ε4 carriers, higher PA levels, measured using self-report [Stanford Brief Activities Survey (SBAS)], were associated with greater functional MRI semantic memory activation [mean age: 72.8(4.89)] (Smith et al., 2011), and when measured by V̇O2 max, higher fitness was associated with greater regional PET cerebral glucose uptake [mean age: 62.67(5.54)] (Deeny et al., 2012). Among *APOE* ε4 carriers only, higher self-reported PA measures (SBAS) were associated with preserved hippocampal volume [mean age: 72.85(4.89)] and hippocampal integrity [mean age: 72.95(4.89)] (Smith et al., 2014; Woodard et al., 2012). Among studies reporting on activity time, one found that low physically active (<3x/week) *APOE* ε4 carriers exhibited lower right temporal activation compared with highly physically active (≥3x/week) carriers [mean age: 59.5(5.0)] (Deeny et al., 2008). Another study that included participants between the ages of 52–81 [mean age: 68.97(8.54)] reported that greater sedentary time (measured by accelerometry) was associated with increased resting left hippocampal cerebral blood flow (CBF) among *APOE* ε4 carriers only (Zlatar et al., 2014). This counterintuitive perfusion finding may reflect compensatory or dysregulated cerebrovascular activity rather than a uniformly beneficial response, underscoring the need to interpret CBF changes in relation to cognitive and pathological endpoints. Functional connectivity associations between higher PA and decreased left temporal lobe synchrony were also observed [mean age: 60.48(8.10)] in both carriers and non-carriers, contributing to preserved brain structure in carriers, and to cognitive function improvements in working and episodic memory domains in non-carriers (De Frutos-Lucas et al., 2020a). The remaining studies which included combined sample age ranges between 40 and 92 years did not find a moderating effect of *APOE* status on PA and imaging outcomes [mean age: 77.3 (3.4), 58.58 (6.33)] (Corlier et al., 2018; Boots et al., 2015) or reported variable findings [mean age: 74.09 (4.62)] (Smith et al., 2016).

#### Primary studies of adults with mild cognitive impairment or dementia at baseline

##### Cognition outcome(s)

Primary studies including either an MCI or dementia sample at baseline (*N* = 6), were all RCTs. Of these RCTs, three studied cognitive outcomes and consistently reported that *APOE* ε4 did not moderate the effect of PA on cognitive function (Eggermont et al., 2009; Karssemeijer et al., 2019; Sanders et al., 2020). These studies that found no effects of physical activity, included older samples with mean ages of 85.4 and 82.3 (SD: 7.0) years, respectively (Eggermont et al., 2009; Sanders et al., 2020). One of the three studies which included a sample mean age of 79.2 (SD: 6.9) reported that, independent of *APOE* genotype, aerobic exercise and exergame training were associated with improved psychomotor speed (Karssemeijer et al., 2019).

##### Fluid/neuroimaging biomarker outcome(s)

The remaining RCTs (*N* = 3) focused on neurodegenerative biomarker outcomes, with peripheral biomarkers such as plasma interferon-gamma levels (IFNγ) and serum brain-derived neurotrophic factor (BDNF). Central biomarkers studied were CSF-based and included Aβ (e.g., Aβ38, Aβ40, and Aβ42), tau (e.g., T-tau, P-tau), soluble amyloid precursor protein (sAPP), and soluble triggering receptor expressed on myeloid cells 2 (sTREM2). In a subgroup analysis of *APOE* ε4 carriers [mean age: 70.60(7.45)], a modulating effect was described, with *APOE* ε4 carriers in the control arm showing increased plasma interferon-gamma (IFNγ) levels and decreased CSF sTREM2 compared to carriers in the aerobic exercise arm (Jensen et al., 2019). In contrast, among a sample of 22 participants with MCI [ε4+, *n* = 9; mean age: 72.00(7.2)], non-carriers demonstrated significant increases in serum BDNF levels after 6 months of aerobic exercise, whereas carriers did not (Allard et al., 2017). The third RCT which assessed CSF AD biomarkers (Aβ, t-tau, p-tau, sAPP) over a 4-month period reported no physical activity associations or *APOE* ε4 modifying effects [mean age: 68.66(5.49)] (Steen Jensen et al., 2016).

#### Primary studies of adults with mixed cognitive status

##### Cognition outcome(s)

One observational study using data from three longitudinal cohort studies [mean cohort ages: 69.8(8.1), 72.4(7.2), 67.8(7.4)] including cognitively unimpaired or MCI adults at baseline focused on cognitive function across a sample of 7,176 adults (ε4+, *n* = 1863) and did not find an interaction between *APOE* ε4 and PA on cognitive decline. Overall, PA was also not significantly associated with cognitive decline in the total sample, though results were heterogeneous across cohorts (Stringa et al., 2020).

One study assessed dementia risk in a sample of 942 adults (ε4+, *N* = 206) above the age of 60 [mean age: 75.85 (7.24)] with unimpaired cognition (*N* = 506) or dementia (*N* = 436) at baseline, matching cases and controls by age within a 5 year-interval, and found a significant association between high-frequency leisure PA and reduced dementia risk, with stronger protective effects for *APOE* ε4 carriers (Yang et al., 2015).

In adults without cognitive impairment, with MCI, or with AD at baseline, three primary studies were identified, and all focused on cognitive outcomes of dementia. One of these longitudinal studies which included a sample of 1,511 adults reported an association between increased midlife PA and a reduced risk of dementia, with more pronounced benefits for males and overweight individuals, and primarily among *APOE* ε4 non-carriers (Tolppanen et al., 2015). The other longitudinal study (*n* = 1,284; ε4+, *n* = 452) indicated that risk factors such as physical inactivity, high dietary fat and alcohol intake, and smoking in midlife were associated with an increased risk of dementia and AD, especially for *APOE* ε4 carriers (Kivipelto et al., 2008). These longitudinal studies were both conducted using participants from the Cardiovascular Risk Factors, Aging and Dementia (CAIDE) cohort, with a mean baseline age of 50.6 (6.0) years during midlife. Although each examined different factors related to the development of dementia later in life, their findings suggest that modifiable factors may have differential effects on *APOE* ε4 carriers compared to non-carriers. The third observational study which indicated cognitive function as a main outcome of interest reported that PA did not correlate with dementia risk, irrespective of *APOE* status (Carlson et al., 2008).

##### Fluid/neuroimaging biomarker outcome(s)

Primary studies, all of which were observational, enrolled cognitively unimpaired, MCI and/or AD adults at baseline (*N* = 7). Of those identified, three studies assessed biomarker outcomes, with two observing no association between PA and neurodegenerative biomarker trajectories such as amyloid deposition and hippocampal volume, regardless of *APOE* status (Vemuri et al., 2016; De Souto Barreto et al., 2015). Both studies that observed no association between PA, *APOE* ε4, and neurodegenerative biomarkers included participants above the age of 70, with mean ages of 78.6 (5.0) and 74.7 (4.2) respectively. One cohort study of 287 cognitively unimpaired or MCI adults [ε4+, *n* = 66; mean ages: 71.91 (6.64)] reported that higher midlife PA appeared to moderate the association between Aβ accumulation and cerebral glucose metabolism, with Aβ accumulation *APOE* ε4 genotype associations (Jeon et al., 2020). Of the remaining two studies, one was an imaging-focused study (*n* = 80) and revealed that increased PA levels were associated with greater functional connectivity between the ventral posterior cingulate cortex (PCC) and the supplementary motor area (SMA) for *APOE* ε4 carriers only [ε4+, *n* = 22; mean ε4 carrier age: 72.18 (5.79)] (Kerestes et al., 2015). The other observational study, which assessed neuroimaging outcomes in a sample of 1,443 adults without dementia at baseline [mean age: 77.2 (6.4)], observed an association between low and middle PA levels and larger hippocampal volume among carriers only (ε4+, *n* = 394). However, *APOE* genotype did not significantly affect the association between PA and total brain volume (TBV) (Gu et al., 2020).

In adults with normal cognition or AD at baseline, three observational primary studies were identified, two of which focused on imaging outcomes. Neither of these studies reported a significant interaction between *APOE* status and PA measures on brain volumetrics (Braskie et al., 2014; Honea et al., 2009). These studies included older samples on average [mean age: 80.54 (5.08), 73.80 (6.3)], respectively.

## Discussion

**Figure 3:**
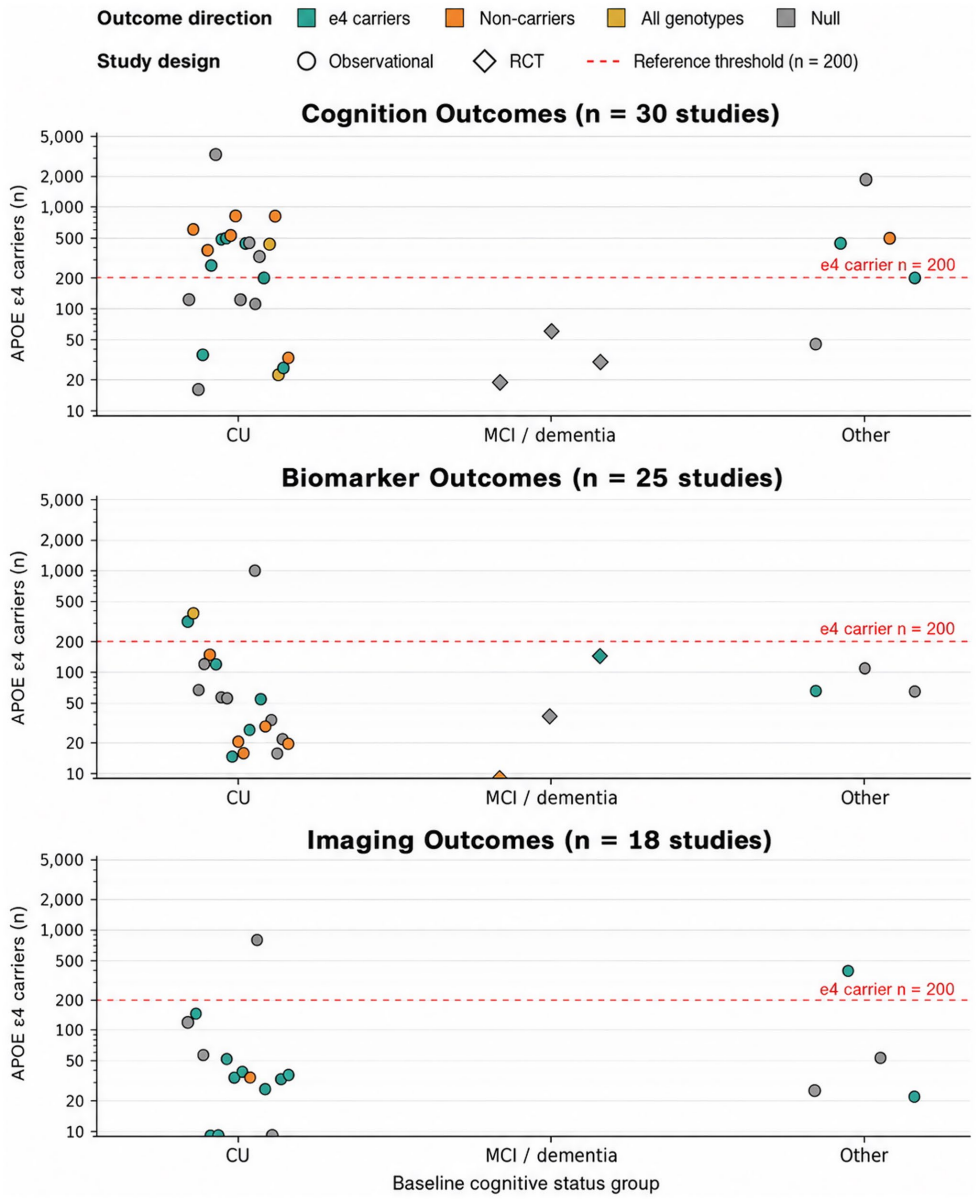
Primary Genotype Study Data of Physical Activity and Cognitive, Biomarker, and Neuroimaging Outcomes. This figure describes observed *APOE* genotype effects across individual PA studies by participant cognition at baseline, reviewing outcomes of cognition, biomarkers, and imaging. Cognitive status groupings at baseline include cognitively unimpaired (CU), mild cognitive impairment (MCI) / dementia, and other (participants with varied cognition samples). Results were categorized by genotype carrier status as well as study design. Indicated effects by *APOE* category include both beneficial responses to physical activity (PA) and adverse effects linked to inactivity. A sum of 63 unique primary studies were included in the figure; studies with more than one relevant outcome domain are plotted once per domain. Five primary studies were omitted from this figure because participant cognitive status at baseline was not explicitly reported and could not be inferred (Espeland et al., 2017; Niti et al., 2008; Gelber et al., 2012; Schuit et al., 2001; Gustavsson et al., 2012). *APOE* status classifications were assigned independent of the study outcome direction.

The individual studies across systematic reviews included in this umbrella review indicate that PA-related cognitive, biomarker, neuroimaging, and vascular/metabolic findings vary by baseline cognitive status and study design. Figure 3 summarizes this pattern across the literature by organizing *APOE*-stratified findings according to cognitive stage, outcome domain, genotype group, and study type. Favorable associations were most often reported in observational studies of younger or cognitively unimpaired adults, whereas randomized trials were fewer, generally enrolled older adults with MCI or dementia, included small *APOE* ε4 subgroups, and reported largely null or mixed genotype-specific effects. Taken together, these findings show that disease stage and study design are central to interpreting the mixed PA–*APOE* ε4 literature.

### Relationship between PA and cognitive, biomarker, and neuroimaging outcomes

**Figure 4:**
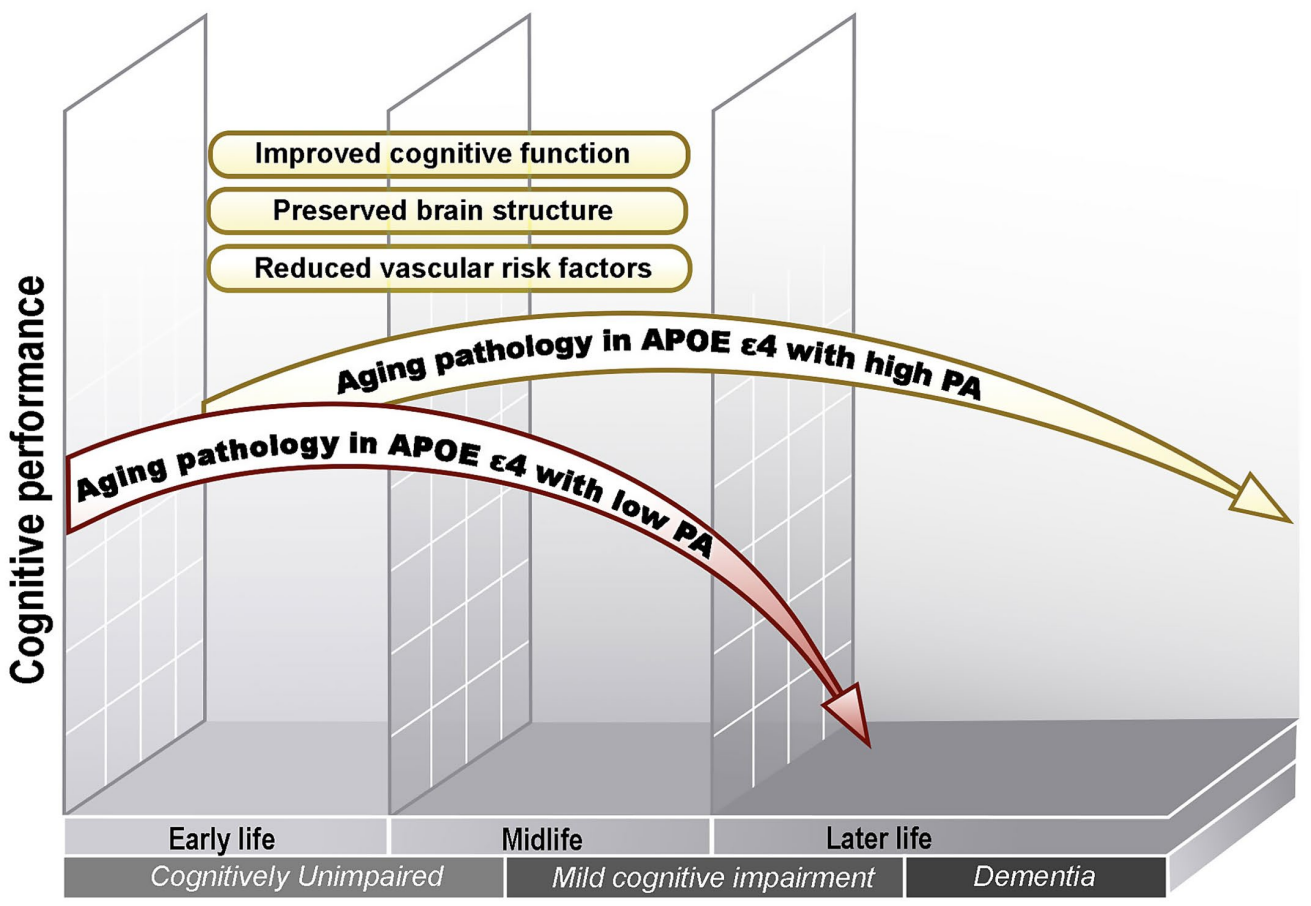
Model of protective effects of physical activity on age-related pathology in *APOE* ε4 carriers across the lifespan.

The stage-specific pattern observed across studies may reflect greater responsiveness to PA earlier in the disease continuum, when cognitive reserve is higher and vascular, metabolic, inflammatory, and neuroplastic pathways may remain more modifiable. However, biological timing cannot be separated from methodological differences across the current literature. The available data therefore support a hypothesis-generating interpretation: PA-related associations may vary by disease stage and *APOE* ε4 status, but the evidence does not establish a uniform or causal genotype-specific effect (Figure 4).

Observational studies reported more consistent associations between increased cumulative PA exposure during midlife and improved cognitive, biomarker, and neuroimaging outcomes in relatively younger and cognitively unimpaired *APOE* ε4 carriers. These studies largely reported that midlife PA is associated with improved memory, executive function, and reduced dementia risk among carriers (Rovio et al., 2005). Moreover, regular midlife PA, which in some studies reflected leisure-time activity and in others structured aerobic exercise, was associated with more protective cognitive outcomes in later life (Kulmala et al., 2014). Meanwhile, older adults or those with cognitive impairment demonstrated less consistent associations, with *APOE* ε4 effects describing mixed results of cognitive, biomarker, and neuroimaging responses to PA exposure. Herein, reviewed RCTs involving participants with MCI or AD on cognitive outcomes included adults with a cumulative mean age of 82.3 years and consistently reported that PA did not affect cognition, and *APOE* genotype did not moderate the relationship between PA and cognitive function (Eggermont et al., 2009; Karssemeijer et al., 2019; Sanders et al., 2020). Neuroimaging findings provide further context for these observed patterns. Studies involving older adult or AD samples at baseline reported no association or interaction between *APOE* status and PA measures on brain volumetrics (Braskie et al., 2014; Honea et al., 2009). Cognitively unimpaired samples, however, predominantly presented with associations between neuroimaging outcomes, *APOE* status, and PA. Among *APOE* ε4 carriers, higher levels of PA were associated with potentially protective neuroimaging profiles, including lower amyloid deposition, greater semantic memory activation, and preserved hippocampal structure/volume (Smith et al., 2011; Brown et al., 2013; Head et al., 2012; Smith et al., 2014; Woodard et al., 2012).

With advancing age, as observed across reviewed studies, individuals tend to progress to more advanced stages of MCI or AD, which may in turn limit their responsiveness to PA intervention. AD and MCI conditions are characterized by progressive neuronal loss, synaptic dysfunction, and brain atrophy (Griffiths and Grant, 2023; Mueller et al., 2010; Devanand et al., 2007; Pini et al., 2016), particularly in the hippocampus and prefrontal cortex, regions that correspond with memory and executive functions. Previous research has associated the *APOE* ε4 allele with accelerated neurodegenerative processes compared to non-carriers (Ng et al., 2025; Manning et al., 2014; Mori et al., 2002; Singh et al., 2023). A recent meta-analysis of voxel-based morphometry studies (*N* = 1,135) examined the role of *APOE* ε4 in brain atrophy, reporting significantly greater hippocampal atrophy among *APOE* ε4 carriers with MCI and AD compared to non-carriers, while cognitively unimpaired controls who were also carriers showed no atrophy in these regions (Bailey et al., 2024).

These patterns are consistent with the hypothesis that earlier intervention confers greater benefit, but they do not establish a simple dose–response relationship. Several randomized trials in cognitively unimpaired adults that were not included provide additional context. The FINGER multidomain lifestyle intervention (*n* = 1,260, ages 60–77), which included exercise alongside dietary guidance, cognitive training, and vascular-risk monitoring, reported genotype-specific cognitive benefits, with significant improvements on neuropsychological testing (estimate 0.037; 95% CI 0.001–0.073; *p* = 0.045) and more specifically on memory (estimate 0.070; 95% CI 0.006–0.135; *p* = 0.03) for *APOE* ε4 carriers but not non-carriers (Ngandu et al., 2015). Additionally, a 52-week aerobic exercise trial in hypertensive participants (ages 65–87) showed improved hippocampal blood flow specifically in *APOE* ε4 carriers (+4.09 vs. − 2.08 mL/100 g/min; *p* = 0.006) (Kaufman et al., 2021). These findings should be interpreted alongside observational data showing increased hippocampal CBF with sedentary behavior in *APOE* ε4 carriers; together, they suggest that perfusion changes may differ by exposure type, intervention context, and disease stage, and may not map linearly onto benefit without concurrent cognitive or pathological endpoints. These trials, while limited in scope, indicate that PA or multidomain lifestyle interventions can produce genotype-specific effects in older at-risk adults, and that null findings in other RCTs likely reflect cohort differences or design limitations rather than true absence of effect. Prospective trials enrolling cognitively unimpaired *APOE* ε4 carriers in midlife, with prespecified biomarker endpoints and adequate follow-up, are needed to test this hypothesis directly.

### Which outcomes should future PA interventions use?

The reviewed studies used heterogeneous cognitive, biomarker, neuroimaging, and vascular/metabolic outcomes, limiting mechanistic interpretation. Future PA studies in *APOE* ε4 carriers may be most informative when outcomes are matched to the disease stage and hypothesized pathway. In cognitively unimpaired or earlier-stage adults, proximal outcomes such as objective PA, cardiorespiratory fitness, lipid profiles, blood pressure-related markers, inflammatory markers, selected neuroimaging measures, and blood-based AD biomarkers may be more feasible and informative than long-term clinical conversion outcomes alone.

Younger, cognitively unimpaired *APOE* ε4 carriers presented with associations between PA and lower amyloid brain deposition compared to studies conducted in older samples with MCI or dementia. This is consistent with previous research which describes a moderating effect of *APOE* ε4 on the relationship between PA and Aβ accumulation in a relatively younger sample (Pedrero-Chamizo et al., 2024), with age-related variability in findings between carriers and non-carriers across several studies (De Frutos-Lucas et al., 2020b).

Further, the use of sensitive, validated peripheral blood biomarkers (e.g., pTau217), which can detect more subtle disease-related changes, may allow for longitudinal assessments that are not practical with CSF biomarker assessments (Georgakas et al., 2023). A prospective cohort study with an average follow-up of 5.65 years after plasma collection demonstrated the prognostic utility of pTau217 in predicting clinical progression to MCI in cognitively unimpaired adults (*n* = 215), with performance comparable to PET and CSF measures (Yakoub et al., 2025). Other studies have also reported correlations between pTau217, PET (e.g., Aβ), and cognitive performance outcomes in cognitively unimpaired samples at baseline (Jonaitis et al., 2023; Rissman et al., 2024; Janelidze et al., 2021). These findings indicate that pTau217 may provide a sensitive and scalable blood-based measure for preclinical AD studies of PA, cognitive decline, and *APOE* status, potentially reducing reliance on PET imaging as the field continues to validate blood-based biomarkers. Recent longitudinal data from cognitively unimpaired older adults further supports this approach. In a cohort with pedometer-measured physical activity, baseline amyloid PET, longitudinal tau PET, and up to 14 years of cognitive follow-up, higher PA was associated with slower amyloid-related cognitive and functional decline (Yau et al., 2025). These associations were not explained by lower baseline or longitudinal amyloid burden, but were mediated by slower inferior temporal tau accumulation. Although this study was not designed to test *APOE* ε4-specific effects, it illustrates the value of objective PA measurement, biomarker enrichment, longitudinal tau-sensitive outcomes, and practical activity targets for future PA studies in earlier-stage at-risk adults. Plasma pTau217 can therefore be prospectively incorporated as part of a broader mechanistic endpoint in PA trials targeting cognitively unimpaired *APOE* ε4 carriers, enabling detection of biological associations before cognitive decline is measurable.

### Mediators of lipid metabolism and inflammatory markers in *APOE* ε4

Although AD-related biomarkers may help clarify whether PA influences amyloid- or tau-associated disease progression, they do not capture the full range of pathways through which PA may affect brain health in *APOE* ε4 carriers. The studies reviewed here suggest that vascular and metabolic outcomes, particularly lipid profiles, may offer more proximal and practical measures of PA response. These pathways are especially relevant because *APOE* plays a central role in lipid transport and cholesterol metabolism. Older adults with MCI or AD have an increased risk of vascular disease, insulin resistance, and systemic inflammation, which may impair the potential cerebrovascular and metabolic benefits of PA. The *APOE* ε4 allele has been associated with mitochondrial dysfunction and alterations in glucose and lipid metabolism, including changes in glycolysis and greater reliance on fatty acids and lipid accumulation (Lysaker et al., 2026). *APOE* ε4 is also linked to altered lipid transport and clearance, including lower plasma *ApoE* levels and dysregulated lipid processing, contributing to lipid accumulation and disrupted lipid homeostasis (Al-Ghraiybah et al., 2026). These changes may influence lipid storage and processing in younger carriers, in turn affecting their response to PA-induced changes in lipid metabolism.

Compared to non-carriers, cholesterol concentrations (LDL-C, TC) have been reported to be elevated among *APOE* ε4 carriers (Belloy et al., 2019; Martins et al., 2006). Previous research has also shown increased blood pressure variability to predict future longitudinal increases in cerebral amyloid retention, tau-mediated neurodegeneration (Sible and Nation, 2022a; Sible et al., 2022), hippocampal atrophy, and entorhinal cortical thinning (Sible and Nation, 2022b) for *APOE* ε4 carriers in particular. No primary studies in this review assessed vascular outcomes in response to PA among MCI or AD samples. However, a number of primary studies with young adult samples focused on lipid metabolism outcomes, and generally reported more protective effects of PA on lipid profiles among *APOE* ε4 carriers (Bernstein et al., 2002; Boer et al., 1998; Corella et al., 2001; Lee et al., 2018; Pisciotta et al., 2003). Among young *APOE* ε4 samples with high PA engagement, improvements in LDL-C, HDL-C, and TG levels were observed (Bernstein et al., 2002; Boer et al., 1998; Corella et al., 2001; Pisciotta et al., 2003). Decreased systolic blood pressure among young *APOE* ε4 adults was also reported after 6 months of exercise training in a sample of 28 (ε4+, *n* = 15) patients with histories of stroke (Lee et al., 2018). These findings suggest potential protective effects of PA on vascular factors among young *APOE* ε4 carriers. These lipid improvements are mechanistically plausible given the established effects of genotype on lipid transport and cholesterol clearance, and are clinically relevant given the elevated cardiovascular risk in this group. However, the reviewed studies do not establish whether PA-induced lipid changes translate into lower AD risk or delayed cognitive decline. Future studies testing vascular or metabolic mechanisms should use outcomes aligned with that pathway, for example by pairing lipid profiles, blood pressure, insulin resistance, or fitness measures with cerebral blood flow, vascular function, hippocampal structure, or tau-related measures, rather than relying on amyloid biomarkers or clinical conversion alone. Inflammatory and neurotrophic markers, including BDNF, may also help characterize PA response, but the reviewed evidence was limited, inconsistent, and based largely on small or older cognitively impaired samples.

### Limitations

The findings of this umbrella review are subject to the limitations of the included systematic reviews and the extracted primary studies. These limitations include methodological heterogeneity, reliance on self-reported PA in most studies, limited use of objective PA measures (e.g., accelerometry), variability in PA definitions and neuropsychological measures, and inconsistent ascertainment of cognitive, biomarker, neuroimaging, and vascular/metabolic outcomes. Confounding factors including sex differences, vascular risk factors, and level of education may also influence the relationship between PA and cognitive, biomarker, and neuroimaging responses. All primary studies of cognitively unimpaired participants included in this umbrella review were observational and therefore cannot establish causality. Randomized trials are few, typically small, and often conducted in older adults with established impairment. Figure 3 was designed as a descriptive visual summary of the *APOE*-stratified primary study evidence; because of heterogeneity in study design, sample size, outcome definitions, *APOE* subgroup reporting, and availability of effect estimates, it should not be interpreted as a weighted synthesis of study quality, sample size, or effect magnitude. Given the umbrella review design and the large number and heterogeneity of the included studies, a systematic evaluation of these factors across primary studies was not feasible. In addition, *APOE* ε4-stratified analyses were often secondary or *post hoc*, raising concerns about multiple comparisons, selective reporting, and limited statistical power within genotype subgroups.

## Conclusion

This umbrella review highlights a stage- and design-dependent pattern in the PA–*APOE* ε4 literature. Favorable associations were most often reported in observational studies of younger or cognitively unimpaired adults, whereas randomized trials were fewer, generally conducted in older adults with MCI or dementia, and often underpowered for *APOE* ε4-specific effects. The current evidence does not establish a definitive genotype-specific preventive effect of PA, but it suggests that PA-related cognitive, biomarker, neuroimaging, and vascular/metabolic findings should be interpreted in relation to disease stage, participant selection, and outcome measurement. Future studies may be most informative if they target earlier-stage, low-active or metabolically at-risk *APOE* ε4 carriers using objective PA measures and proximal vascular, metabolic, imaging, or blood-based biomarker outcomes. This approach may provide a more practical path for determining whether PA has biologically meaningful effects in genetically at-risk populations before clinical decline is expected.

## Footnotes

1
Covidence.org
