# Supplementary material for: APOE ε4, physical activity, and the brain: a review of systematic reviews
Source: Front Aging Neurosci. 2026 Jul 23;18:1798639. doi: 10.3389/fnagi.2026.1798639 (PMC13442396; doi:10.3389/fnagi.2026.1798639)
Supplement: Supplementary file 1 [file Data_Sheet_1.docx]

**EXERCISE PAPER MASTER TABLE LIST**

*Table 1. Cognitively Normal (CN) Studies* …………………………………………………………… **2-7**

Primary Studies Excluded:

- Abbott et al., 2004
- Buchman et al., 2012
- Larson et al., 2006
- Lindsay et al., 2002
- Paillard-Borg et al., 2009
- Rovio et al., 2007
- Wilson et al., 2002

*Table 2. Mild Cognitive Impairment (MCI), Dementia Studies* ………………………………………**8-9**

Primary Studies Excluded:

- Cheng et al., 2012

*Table 3. Mixed Cognitive Status (i.e., CN or MCI; CN or AD; CN, MCI, OR AD)* ………………….**9-10**

Excluded:

- Wang et al., 2006

*Table 4. Cognitive Status Unspecified (NS) Studies* ………………………………………………….**11-12**

Primary Studies Excluded:

- de Brujin et al., 2013

| **Table 1. CN (ALL OBSERVATIONAL; n=43)** | | | | | | | | | | |
| --- | --- | --- | --- | --- | --- | --- | --- | --- | --- | --- |
| **Citation** | **Follow-up** | **Age** | **Sex** | **Sample** | **Study Design** | **PA Assessment** | **Main Outcome(s)** | | ***APOE* Results** | **Main Results** |
| Abbott (2004)^1^ | ~ 7 y | 71-93 y | Male | **ε4+:** 365  **Total:** 2257 | Prospective Cohort | Self-reported walking (mi./day) | Overall dementia, AD, VaD | NS | | Walking <2 mi./day vs. >2 mi./day associated with a nonsignificant lowered dementia risk. |
| Boer (1997)^2^ | N/A | 18 - 26 y | Male Female | **ε4:** 381  **Total:** 1448 | Cross-sectional | Self-reported questionnaire | TC | No association between *APOE* genotype and PA. | | PA uniformly influenced lipid levels, with no presence of an *APOE* interaction. |
| Boots (2015)^3^ | N/A | 40-65 y  58.58 ± 6.33 | Male Female | **ε4:** 122  **Total:** 315 | Cross-sectional | Non-exercise cardiorespiratory fitness  Sub-set performed Graded Exercise Test | GM volumes,  WMH, cognitive function | *APOE* ε4 did not moderate associations between CRF, GM volumes, WMH, or cognitive outcomes. | | Higher PA associated with improved brain structure, cognitive performance, and reduced WMH. |
| Brown (2013)^4^ | N/A | 60-95 y  69.6 ± 6.8 | Male Female | **ε4+:** 148  **Total:** 546 | Cross-sectional | International Physical Activity Questionnaire | Amyloid deposition, plasma Aβ | High PA associated with lower plasma Aβ only in non-carriers.  Higher PA levels associated with lower amyloid brain deposition (PiB PET) in *APOE* ε4 carriers only. | | Higher PA levels associated with higher HDL, lower insulin, and TG levels. |
| Buchman (2012)^5^ | ~ 4 y | ≥ 65 y | Male  Female | **ε4+:** NS  **Total:**  716 | Prospective cohort | Wrist actigraphy ≤10 days | Daily PA, AD incidence | *APOE* ε4 status did not attenuate association of PA with incident AD. | | Total daily PA associated with incident AD. |
| Chang (2010)^6^ | ~ 26 years | (Midlife Examination: 51, AGES examination: 76) | Male  Female | **ε4+:** 583  **Total:** 4945 | Longitudinal cohort | Interview (categorized by h/week) | Cognitive function (processing speed, memory, executive function), dementia risk | Sub-sample analysis revealed sedentary mid-life *APOE* ε4 carriers had an increased risk for dementia compared to non-carriers. | | Regular mid-life PA associated with better late-life cognitive function (SP, MEM, EF), and lowered dementia risk at follow-up. |
| Corlier (2018)^7^ | ~ 9 years | ≥65 years  77.3 ± 3.4 | Male Female | **ε4+:** 57  **Total:** 335 | Longitudinal | Minnesota Leisure Time Physical Activities Questionnaire | Regional cortical thickness, serum CRP | *APOE* ε4 carriers had lower CRP levels and cortical thickness compared to non-carriers. | | Higher PA associated with greater hippocampal volume.  High serum CRP at baseline associated with decreased cortical thickness at follow-up. |
| de Frutos-Lucas (2018)^8^ | N/A | > 65 y  71.84 ± 4.28 | Male  Female | **ε4+:** 20  **Total:** 100 | Cross-sectional | International Physical Activity Questionnaire | Neurophysiological marker (IAPF) | PA positively associated with higher IAPF in non-carriers. | | High levels of PA associated with increased IAPF values. |
| de Frutos-Lucas (2020)^9^ | N/A | 50-82 y  60.48 ± 8.10 | Male Female | **ε4:** 33  **Total:** 107 | Cross- sectional | Accelerometry (ActiGraph GT3X+) for 7 days (≥10 h/day) | Functional Connectivity | FC associated with preserved brain structure (GM, WM) in ε4 carriers, and to improved cognition (working and episodic memory) in non-carriers. | | PA was associated with reduced FC in the left temporal lobe in both *APOE* ε4 carriers and non-carriers. |
| de Frutos-Lucas (2020)^10^ | N/A | 48-82 y  59.92 ± 7.52 | Male Female | **ε4:** 36  **Total:** 113 | Cross- sectional | Accelerometry (ActiGraph GT3X+) for 7 days (≥10 h/day) | Resting-state alpha band power (MEG) | In older *APOE* ε4 carriers, increased alpha power associated with improved memory and brain structure preservation (GM and precuneus volume). | | PA positively associated with alpha power in a parieto-occipital cluster in younger ε3 homozygotes and older ε3/ ε4 carriers. |
| Deeny (2008)^11^ | N/A | 50-70 y | Male Female | Sternberg analysis  **ε4+:** 16  **Total:** 54  MEG analysis  **ε4+:** 9  **Total:** 23 | Cross-sectional | Yale Physical Activity Survey | Imaging, Cognition (Sternberg working memory task) | Post-hoc analysis showed sedentary carriers had lower right lobe temporal activation compared to active carriers and non-carriers. | | Interactive effect of high PA and *APOE* ε4 on RT.  Neuropsychological exam not significantly predicted by *APOE* genotype. |
| Deeny (2012)^12^ | N/A | 62.67 (Carriers: 63.7 (6/37), non-carriers: 61.7(4.72)) | Female | **ε4+:** 9  **Total:** 18 | Cross-sectional | Yale Physical Activity Questionnaire  Maximal fitness test | Cerebral metabolism (VO_2_ max, CVLT and FDG-PET) | High PA *APOE* ε4 carriers had greater cerebral glucose uptake than low PA carriers in the temporal lobe, while low PA carriers had greater uptake in frontal and parietal lobes. | | Significant effect of PA level on FDG-PET during working memory for *APOE* ε4 carriers only. |
| Etnier (2007)^13^ | N/A | 51-81 y  62.00  ± 7.39 | Female | **ε4+:** 35  **Total:** 94 | Cross-sectional | Aerobic fitness test (graded exercise test) | Cognition (AVLT, CFT, WCST) | Significant association between aerobic fitness and memory performance for *APOE* ε4 homozygotes, but not heterozygotes or non-carriers | | Aerobic exercise is positively associated with memory improvement for *APOE* ε4 homozygotes. |
| Fenesi (2017)^14^ | ~ 5 years | ≥65 years | Male  Female | **ε4+:** 376  **Total:** 1646 | Population- based cohort | PA interview (categorized as exercisers and non-exercisers) | Dementia incidence | Significant interaction between *APOE* status and PA on dementia risk, with low PA non-carriers at greater risk than high PA non-carriers. | | Low PA associated with higher dementia risk compared to high PA for non-carriers only. |
| Ferrari (2013)^15^ | ~ 9 y | ≥ 75 y  (Non-carriers 80.5 (4.7), Carriers 80.1 (4.2)) | Male  Female | **ε4+:** 265  **Total:** 932 | Prospective cohort | Leisure activities personal interview | Education level, vascular risk factors, PA level, dementia incidence | *APOE* ε4 significantly associated with increased dementia risk and AD.  Additive interaction of high education, absence of vascular factors, and high leisure activity with *APOE* ε4 on dementia risk.  More pronounced association of higher leisure PA and lower AD incidence among *APOE* ε4 carriers. | | PA significantly associated with reduced AD risk. |
| Hansson (2019)^16^ | Vasaloppet  ~ 21 y  MDCS  ~ 18 y | Vasaloppet: Median age 36.0 years  MDCS: Median age 57.5 years | Male  Female | MDCS  **ε4+:** 3306  **Total:** 20,639  Vasaloppet  **ε4+:** NS  **Total:**  395,369 | Prospective | MDCS  Self-reported questionnaire (engagement in leisure time PA)  Vasaloppet Skier index race (non-skier controls) | VaD; AD incidence | *APOE* ε4 did not moderate the association between mid-life PA and dementia incidence. | | Higher PA associated with a reduced incidence of VaD, but not AD nor all-cause dementia. |
| Head (2012)^17^ | N/A | 45-88 y | Male  Female | PET analysis  **ε4+:** 52  **Total:** 163  CSF analysis  **ε4+:** 56  **Total:** 165 | Cross-sectional | Self-report questionnaire of WRJ activity | CSF Aβ_42_, amyloid deposition imaging (PET) | Less reported PA associated with higher PIB for carriers only.  Association between *APOE* ε4 status and lower amounts of CSF Aβ_42._ No interaction between *APOE* and PA on CSF Aβ_42._ | | Significant interaction between *APOE* and PA on cerebral amyloid deposition (PiB-PET). |
| Krell-Roesch (2016)^18^ | ~3.2 y (1.9y-4.7y) | 70-93 y | Male  Female | **ε4:** 474  **Total:** 1830 | Prospective cohort | Minnesota leisure time physical activity questionnaire  1985 National Health Interview Survey | Incident MCI | Inactive *APOE* ε4 carriers had an increased risk of MCI vs. active non-carriers. | | Significant additive interaction between mid- and late-life PA in lowered risk of MCI.  Light and vigorous PA in mid-life associated with reduced risk of incident MCI.  Late-life light and moderate PA associated with reduced risk of incident MCI. |
| Kulmala (2014)^19^ | ~ 20.1 y | 65-79 y | Male  Female | **ε4+:** 484  **Total:** 1511 | Prospective cohort | Self-reported questionnaire. | All-cause dementia | Decreased PA from mid- to late life associated with dementia risk, with more pronounced effects in *APOE* ε4 carriers. | | Poor PA at mid-life associated with increased dementia risk in *APOE* ε4 non-carriers, males, and those with chronic conditions. |
| Larson (2006)^20^ | ~ 6.2 y | ≥65 y | Male  Female | **ε4+:** 390  **Total:** 1740 | Prospective cohort | Self-report questionnaire (# of days/week engaged in 15 min. of exercise or >) | Dementia incidence, AD, PA level | NS | | PA engagement of more than 3x/week associated with delayed AD onset. |
| Liang (2010)^21^ | N/A | 55–88 y | Male Female | CSF analysis  **ε4:** 21  **Total:** 56  PiB PET analysis  **ε4:** 16  **Total:** 54 | Cross- sectional | WRJ questionnaire | Biomarkers (PIB, Aβ42, tau, pTau181) | Increased PA associated with lower PIB binding and higher Aβ42 levels, with more pronounced associations in *APOE* ε4 non-carriers. | | Elevated PIB and tau associated with lower PA, controlling for covariates reduced to nonsignificant. |
| Lindsay (2002)^22^ | ~5 y | ≥65 y | Male Female | **ε4+:** NS  **Total:** 4088 | Prospective cohort | Self-report questionnaire [engagement in regular exercise (Yes/No)] | AD incidence | Participants with ≥1 APOE ε4 allele had a 3.28x greater risk of AD vs. ε3 carriers. | | Regular PA significantly associated with a lower risk of AD. |
| Luck (2014)^23^ | ~ 4.5 y | ≥75 y | Male  Female | **ε4+:** 514  **Total:** 2492 | Prospective cohort | Self-report questionnaire (engagement in physical activities) | Dementia; AD risk | Significant individual associations of *APOE* ε4 and decreased PA with an increased AD risk.  Interaction of low number of PA and *APOE* ε4 was associated with higher RR of dementia and AD than presence of one factor alone (possible additive effect). | | Regular PA during late life is associated with reduced AD risk. |
| Paillard-Borg (2009)^24^ | ~9 years | ≥75 y | Male  Female | **ε4+:** NS  **Total:** 776 | Longitudinal | Leisure activities personal interview | Dementia incidence | No significant interaction found between *APOE* ε4 and PA. | | Participation in physical, mental, and social activities was associated with a lower risk of dementia, with more pronounced protective effects for those with higher levels of engagement. |
| Podewils (2005)^25^ | ~ 5.4 y | ≥65 y | Male  Female | **ε4+:** ~813  **Total:** 3375 | Prospective cohort | Modified Minnesota Leisure Time Activity  Questionnaire | Dementia; AD; VaD incidence | Inverse association between PA and dementia risk for  *APOE* ε4 noncarriers, but not for carriers.  No significant interaction of energy expenditure or PA index with *APOE* status. | | Number of physical activities was inversely associated with dementia risk in non-ε4 carriers. |
| Ravaglia (2008)^26^ | ~ 4 y | ≥65 y | Male  Female | **ε4+:** 123  **Total:** 749 | Prospective cohort | Paffenbarger Physical Activity Questionnaire | Dementia; AD; VaD incidence | No significant association between *APOE* ε4 and PA was observed. | | Increased PA reduced the risk of vascular dementia, but not Alzheimer's disease.  VaD risk was significantly lower for the upper tertiles of walking, moderate, and total PA vs. the lowest tertile. |
| Rovio (2005)^27^ | ~21 y | 65-79 y  (midlife 50.8 (6.1), re-examination 71.5 (4.0) | Male  Female | **ε4+:** 438  **Total:** 1449 | Prospective cohort | Self-report questionnaire (engagement in leisure-time PA) | Dementia; AD incidence | Additive interaction between PA and *APOE* ε4 reached borderline significance (RERI=0·45, p=0·062). | | Leisure-time PA (≥2x/week) associated with a reduced dementia and AD risk, with more pronounced benefits for *APOE* 4 carriers. |
| Rovio (2007)^28^ | ~ 21 y | 65-79 y | Male  Female | **ε4+:** NS  **Total:** 1449 | Prospective cohort | Self-report questionnaire (engagement in occupational and commuting PA) | Dementia risk, AD | No interaction or association found between work-related PA and *APOE* ε4 | | Work-related PA not associated with dementia and AD risk. |
| Scarmeas (2009)^29^ | ~5.4 y | ≥65 y  77.2 ± 6.6 | Male  Female | **ε4+:** 443  **Total:** 1880 | Prospective cohort | Godin leisure time exercise questionnaire | Time to incident AD | *APOE* did not moderate the association between PA and AD incidence. | | Higher PA associated with lower AD development. |
| Scarmeas (2011)^30^ | ~5.2 y | ≥65 y  77.8 ± 6.4 | Male  Female | **ε4+:** 110  **Total:** 357 | Prospective cohort | Modified Godin leisure time exercise questionnaire | AD incidence | *APOE* did not moderate the association between PA and AD incidence. | | Compared to inactive participants with AD, participants with much PA or some PA had a significantly lower mortality risk. PA did not significantly affect cognitive function. |
| Shaaban (2019)^31^ | ~ 10 y | ≥ 65 y | Male  Female | **ε4+:** 330  **Total:** 1701 | Prospective longitudinal cohort | Self-reported volume of weekly walking | All-cause dementia incidence | The combined effect of low PA and *APOE* ε4 associated with additively higher dementia risk.  No significant synergistic interaction of *APOE* ε4 with PA. | | Several VCMRFs (vascular and cardiometabolic risk factors) associated with all-cause dementia risk, with more pronounced protective effects among *APOE* ε4 carriers. |
| Shih (2018)^32^ | ≤ 10 y | ≥ 60 y  High PA; 69.2 (6.2), Low PA; 70.5 (7.2) | Male  Female | **ε4:** 201  **Total:** 1438 | Prospective cohort | Compendium of Physical Activities (MET hours/week) | Dementia; cognitive impairment risk | *APOE* ε4 carriers with low PA had a threefold risk of dementia/CIND vs. high PA non-carriers. | | Inverse association between high PA and risk of dementia/CIND. |
| Smith (2011)^33^ | N/A | 65–85 y | Male  Female | **ε4+:** 34  **Total:** 68 | Cross-sectional | Stanford Brief Activity Survey | Brain activation (fMRI), cognition (MMSE) | *APOE* ε4 carriers with high PA associated with greater semantic memory activation compared to low PA carriers and non-carriers. | | Interaction of *APOE* and PA on brain activation patterns. |
| Smith (2014)^34^ | ~18 months | 65-89 y | Male  Female | **ε4+:** 39  **Total:** 97 | Longitudinal Cohort | Stanford Brief Activity Survey | Hippocampal volume | Significant interaction between *APOE* status and PA on hippocampal volume, with decreases for low PA *APOE* ε4 carriers (no interaction observed for non-carriers). | | Protective association of increased PA on hippocampal volume in *APOE* ε4. |
| Smith (2016)^35^ | N/A | 65-89 y | Male Female | **ε4+:** 34  **Total:** 88 | Cross-sectional | Stanford Brief Activity Survey | WM tract integrity | Low PA *APOE* ε4 carriers had lower radial diffusivity compared to high PA carriers.  PA associated with higher FA and lower radial diffusivity in non-ε4 carriers only. | | Interactions between PA and *APOE* status observed among several WM tracts. |
| Stojanovic (2020)^36^ | CSF  ~3.47 y  PET-PIB  ~ 4.14 y  Cognitive function  ~ 6.05 y | **55-88 y** | **Male Female** | CSF analysis  **ε4:** 34  **Total:** 95  PiB PET analysis  **ε4:** 56  **Total:** 181  Cognitive function analysis  **ε4:** 110  **Total:** 327 | Longitudinal | Self-report questionnaire (WRJ activity over past 10 y). | AD biomarkers (Aβ, tau), Cognitive function | *APOE* status did not moderate the effect of PA on AD biomarker or cognition outcomes. | | Greater PA associated with lower declines in processing speed (*time* x *exercise* interaction). |
| Taaffe (2008)^37^ | ~ 6.1 y | 71-92 y | Male | **ε4+:** ~428  **Total:** 2263 | Prospective cohort | Self-report questionnaire (# hrs./day spent in five levels of physical activities) | Dementia incidence | No significant interaction of *APOE* status and PA on overall dementia, AD, and VaD. | | High levels of PA associated with reduced dementia risk, but only for men with low physical function. |
| Tan (2016)^38^ | ~ 10 years | ≥ 60 years  Female (71± 7) Male (70±7) | Male  Female | **ε4+:** 796  **Total:** 3714 | Longitudinal cohort | Physical Activity Index | Incident dementia, TCBV, HPV | No interaction found for *APOE* status x PA.  *APOE* ε4 non-carriers in lowest PAI quintile had increased dementia risk compared to higher PAI quintiles, strongest in ≥75 y. | | Participants in the lowest quintile of PA had a 50% increased risk of incident dementia compared to those in higher quintiles. Linear association between PA and total brain and hippocampal volumes. |
| Tsai (2019)^39^ | N/A | 30-70 y  Carriers (53.19 ± 8.57) Non-carriers (53.94 ± 7.09) | Male Female | **ε4:** 16  **Total:** 33 | Cross- sectional | Senior Functional Physical Fitness Test  Rockport Fitness Walking Test | Molecular Biomarkers,  EEG oscillations | *APOE* ε4 carriers exhibited lower ARs, and an abnormal EEG oscillation on a memory task (4-item condition). | | In *APOE* ε4 carriers, CRF associated with cognitive function.  No significant differences between carriers and non-carriers in regards to biomarkers. |
| Tsai (2021)^40^ | N/A | 38-73 y | Male Female | **ε4:** 22  **Total:** 44 | Cross-sectional | Senior Functional Physical Fitness Test  Rockport Fitness Walking Test | Molecular Biomarkers (Aβ peptides, cytokines, neuroprotective growth factors), cognitive function (ERP) | Significant association between *APOE* ε4 and lower memory condition performance, with smaller ERP P3 amplitudes. | | CRF levels associated with cognitive function, and several molecular biomarkers (Aβ, BDNF).  Carrier group similar to non-carrier group for biomarkers and most measures of neurocognitive performance. |
| Wilson (2002)^41^ | ~ 4.1 y | ≥65 y | Male  Female | **ε4+:** NS  **Total:** 835 | Prospective cohort | Modified 1985 Health Interview Survey | AD incidence | NS | | Weekly hours of engagement in PA did not associate with AD risk. |
| Woodard (2012)^42^ | 18 months | 65-88 y  Stable (72.6 ± 5) Declining (73.6 ± 4.7) | Male  Female | **ε4+:** 26  **Total:** 78 | Prospective cohort study | Stanford Brief Activity Survey | Cognitive function, hippocampal volume, semantic processing activation | Increased PA reduced risk of cognitive decline for *APOE* ε4 carriers only. | | Significant interaction between PA and *APOE* ε4 on cognition. |
| Zlatar (2014)^43^ | N/A | 52-81y | Male Female | **ε4:** 9  **Total:** 33 | Cross-sectional | Accelerometry (7-day period) | Hippocampal CBF | Non-significant association between PA levels and CBF by *APOE* status. | | Association between sedentary time and left hippocampal CBF among *APOE* ε4 carriers only. |

| **Table 2. MCI (n=2), Dementia (n=7)** | | | | | | | | | |
| --- | --- | --- | --- | --- | --- | --- | --- | --- | --- |
| Citation | Follow-up | Age | Sex | Sample | Study Design | PA Assessment | Main Outcome(s) | *APOE* Results | Main Results |
| RCT (n=6) | | | | | | | | | |
| Allard (2017)^44^ | ~ 6 months | 55-89 y | Male  Female | **ε4+:** 9  **Total:** 22  (MCI) | RCT | **Experimental:** aerobic exercise  45-60mn. 3x/week for 6 months.  **Control:** stretch treatment | BDNF serum levels, cardiorespiratory fitness (VO₂ max**)** | Non-ε4 carriers showed significant increases in BDNF levels at 6-months versus carriers. | *APOE* ε4 modulates BDNF response to aerobic exercise, with non-ε4 carriers showing significant increases and ε4 carriers exhibiting a reduced response. |
| Cheng (2012)^45^ | ~ 9 months | ~81.4 ± 7.2 | Male  Female | **ε4+:** ~ 26  **Total:** 110  (dementia) | RCT | **Experimental:**  Tai Chi  60 mn. 3x/week for 12 weeks  **Control:** simple handicrafts | Cognitive function (MMSE) | *APOE* ε4 carriers had lower verbal memory scores and MMSE | Significant moderating effects of TC on MMSE / forward digit span at 9 months. |
| Eggermont (2009)^46^ | ~ 3 months | 85.4 y | Male  Female | **ε4+:** NS  **Total:** 97  (dementia) | RCT | **Experimental:** participants walked for 30 min. 5x/ week for 6 weeks.  **Control:** social visits | Cognitive function (memory, executive function, total cognition) | *APOE* ε4 did not moderate the relationship between PA and cognition (no time×group× *APOE* ε4 interaction effects). | Walking PA did not associate with cognitive function. |
| Jensen (2016)^47^ | ~ 4 months | 50-90 y | Male  Female | **ε4+:** 37  **Total:** 53  (dementia) | RCT | **Experimental:**  60mn. 3x/week mild-to-high intensity PA for 16 weeks.  **Control:** unmonitored PA | CSF AD biomarkers (Aβ, t-tau, p-tau, sAPP) | No effect of *APOE* status on relationship between PA and AD. | No significant effect of PA on AD biomarkers. |
| Jensen (2019)^48^ | ~ 4 months | 50-90 y | Male  Female | **ε4+:** 143  **Total:** 198  (dementia) | RCT | **Experimental:** aerobic exercise (60 min. 3x/week of moderate-to-high intensity for 16 weeks)  **Control:** usual care | Inflammatory markers in plasma and CSF | Sub-group analysis revealed plasma IFNγ increased more in *APOE* ε4 carriers in the control versus carriers in the PA group. | Plasma IL-6 and increase in sTREM2 levels significantly increased in the PA group vs. control. |
| Karssemeijer (2019)^49^ | ~ 6 months | ≥ 60 y  79.2± 6.9 | Male  Female | **ε4+:** 60  **Total:** 115  (dementia) | RCT | **Experimental:** aerobic training or exergame training (3x/week for 12 weeks)  **Control:** relaxation/flexibility | Cognitive function (executive function, episodic memory, working memory, and psychomotor speed) | *APOE* ε4 did not moderate effect of PA on cognitive function. | Significant improvement in psychomotor speed in PA group vs. control group. No significant effect of PA on other cognitive domains. |
| Sanders (2020)^50^ | ~ 9 months | 65-89 y  82.3 ± 7.0 | Male  Female | **ε4+:** 30  **Total:** 69  (dementia) | RCT | **Experimental**: combined aerobic + strength training program (3x/week low-to-high intensity for 24 weeks)  **Control**: flexibility exercises, recreational activities | Physical function, cognitive function (MMSE) | *APOE* ε4 did not moderate the effect of PA on physical or cognitive function. | No significant effect of PA vs. control on measures of physical or cognitive function. |
| Observational (n=1) | | | | | | | | | |
| Carlson (2008)^51^ | 28-36 y | Midlife: 44.7 ± 3.6  Last follow up: (72.7 ± 5.6) | Male | **ε4+:** 46 twin-pairs  **Total:** 147 twin-pairs  (dementia) | Prospective | Self-report questionnaire (engagement in leisure-time PA) | Dementia risk | PA did not predict dementia risk among all twin-pairs, irrespective of *APOE* status. | Mid-life PA did not modify dementia risk. |
| Gu (2020)^52^ | 2.69 y | > 65 y  77.2 ± 6.4 | Male Female | **ε4:** 394  **Total:** 1443  (MCI) | Cross-sectional | Godin Leisure Time Exercise Questionnaire | TBV, cortical thickness, WMH | *APOE* status did not significantly moderate the association between PA and TBV.  Among *APOE* ε4 carriers, low to middle PA levels associated with larger hippocampal volume. | Higher levels of PA associated with larger brain volume. |

| **Table 3. MIXED COGNITIVE STATUS AT BASELINE [CN or MCI (ALL OBSERVATIONAL; n=6); CN or AD (ALL OBSERVATIONAL; n=3); CN, MCI, or AD (ALL OBSERVATIONAL; n=2)]** | | | | | | | | | |
| --- | --- | --- | --- | --- | --- | --- | --- | --- | --- |
| Citation | Follow-up | Age | Sex | Sample | Study Design | PA Assessment | Main Outcome(s) | *APOE* Results | Main Results |
| de Souto Barreto (2015)^53^ | N/A | 70-79 y  74.7 ± 4.2 | Male Female | **ε4:** 65  **Total:** 268  (CN or MCI) | Cross-sectional | Self-report questionnaire (engagement in leisure-time PA) | Aβ deposition | *APOE* status did not moderate the relationship between PA and Aβ deposition. | No association between PA level and Aβ deposition. |
| Jeon (2020)^54^ | N/A | 55-90 y  72 y | Male  Female | **ε4+:** 66  **Total:** 287  (CN or MCI) | Observational cohort | Lifetime Total Physical Activity Questionnaire | Aβ, CGM | Mid-life PA moderated effect of *APOE* ε4 associated Aβ accumulation on CGM.  Inverse association between Aβ retention and CGM at low PA. | Mid-life PA did not moderate the effect of *APOE* ε4 on Aβ accumulation.  Mid-life PA significantly moderated the impact of β-amyloid retention on cerebral glucose metabolism. |
| Kerestes (2015)^55^ | N/A | ≥60 y  Carriers: 72.28  Non-carriers: 72.79 | Male Female | **ε4:** 22  **Total:** 80  (CN or MCI) | Cross- sectional | Pedometer (steps/week) | PCC functional connectivity | *APOE* ε4 carriers had significantly higher dorsal and ventral PCC compared to non-carriers. | Increased levels of PA associated with greater connectivity between the ventral PCC and the SMA in *APOE* ε4 carriers. |
| Stringa (2020)^56^ | ~9 y | ≥ 55 y | Male Female | **ε4+:** 1863  **Total:**  7176  (CN or MCI) | Longitudinal cohort | Self-report questionnaires: (1) LAPAQ, (2) Zutphen Physical Activity Questionnaire, and (3) interview-administered | Cognitive function (MMSE) | No gene-by-environment interaction between *APOE* and PA on cognitive decline. | No association between PA and cognitive decline. |
| Vemuri (2016)^57^ | N/A | 70-89 y | Male Female | **ε4:** 109  **Total:** 393  (CN or MCI) | Longitudinal | Mid-life Physical Activity Questionnaire | Aβ deposition | *APOE* ε4 status positively associated with amyloid deposition. | Mid-life PA did not associate with AD biomarker trajectories. |
| Wang (2006)^58^ | ~ 5.9 y | ≥65 y | Male  Female | **ε4+:** 524  **Total:** 2288  (CN or MCI) | Prospective cohort | Self-report questionnaire (# of days/week engaging in PA ≥ 15 minutes) | Dementia; AD  incidence | NS | Percent of participants with active PA levels was higher (76%) in dementia-free at follow-up vs. participants that developed dementia (66%). |
| Braskie (2014)^59^ | ~ 9 y | ≥ 65 y | Male  Female | **ε4+:** 25  **Total**: 82  (CN or AD) | Case-control | Modified Minnesota Leisure Time Physical Activities questionnaire | Regional brain volume (MRI), blood serum TNFα measures | *APOE* ε4 status significantly associated with smaller total brain volume. | Low serum TNFα and high self-reported PA independently associated with higher total brain volume. |
| Honea (2009)^60^ | N/A | > 65 y  Nondemented (73.3 ± 6.2) Early AD 74.3 ± 6.3 | Male Female | **ε4:** 54  **Total:** 116  (CN or AD) | Cross-sectional | Physical Activity Scale in the Elderly  Graded Exercise Test | GM; WM volume | No interaction between *APOE* status and CRF on GM and WM volumes. | Among AD patients only, CRF associated with larger regional brain volumes. |
| Yang (2015)^61^ | N/A | ≥60 y | Male  Female | **ε4+:** 206  **Total:** 942  (CN or AD) | Case-control | Self-report questionnaire (engagement in leisure-time PA) | AD; VaD risk | Among *APOE* ε4 carriers, high-frequency leisure PA associated with decreased dementia risk.  No significant interaction observed between *APOE* ε4 status and leisure PA on dementia risk. | High-frequency PA (>3x/week) associated with a reduced risk of AD and VaD. |
| Kivipelto (2008)^62^ | ~21 y | 65-79 y  Carriers: 49.6 ± 5.7  Non-carriers (50.3 ± 6.0) | Male  Female | **ε4+:** 452  **Total:** 1284  (CN, MCI, or AD) | Longitudinal | Self-report questionnaire (duration of leisure-time PA) | Dementia; AD incidence | Physical inactivity associated with increased risk of dementia and AD, especially among *APOE* ε4 carriers | Physical inactivity, dietary fat and alcohol intake, and smoking at mid-life associated with risk of dementia and AD, with more pronounced effects among *APOE* ε4 carriers. |
| Tolppanen (2015)^63^ | ~28.3 y | 78.8 y | Male  Female | **ε4+:** 491  **Total:** 1511  (CN, MCI, or AD) | Longitudinal cohort | Self-report questionnaire (engagement in leisure-time PA) | Dementia; AD risk | Absolute risks of dementia were higher in *APOE* ε4 carriers compared with noncarriers among all PA categories.  Lower PA levels among *APOE* ε4 non-carriers associated with a higher dementia risk. | Higher mid-life PA associated with lower dementia risk, with more pronounced protective effects for males, overweight individuals, and *APOE* ε4 non-carriers.  Decreased PA levels from mid-life to late life associated with dementia risk. |

| **Table 4. Cognitive Status Not Specified (n=15)** | | | | | | | | | |
| --- | --- | --- | --- | --- | --- | --- | --- | --- | --- |
| Citation | Follow-up | Age | Sex | Sample | Study Design | PA Assessment | Main Outcome(s) | *APOE* Results | Main Results |
| RCT (n=1) | | | | | | | | | |
| Espeland (2017)^64^ | ~10-13 y | 45-76 y | Male  Female | **ε4:** 724  **Total:** 3802 | RCT | **Experimental:** ≥175 min/week of PA + caloric intake goals  **Control:** education and support sessions | MCI prevalence | Higher MCI prevalence among *APOE* ε4 carriers. | Intervention did not associate with MCI prevalence.  A sub-group analysis  revealed a beneficial intervention effect on cognition for participants with lower baseline BMI, and potential harm for participants with higher baseline BMI. |
| Observational (n=14) | | | | | | | | | |
| Bernstein (2002)^65^ | N/A | 35-74 y | Male  Female | **ε4+:** 320  **Total:** 1708 | Cross-sectional | Self-report questionnaire (% in high-intensity PA) | HDL-C, LDL-C, TG | Male *APOE* ε4 carriers with increased PA (%high intensity) had more protective effects with HDL-C and TG versus non-carriers.  Among female ε4 carriers, there was protective effect of PA on HDL-C levels compared to *APOE* ε2 carriers only. | PA modulates atherogenic effect of *APOE* ε4 on lipid profile. |
| Boer (1998)^66^ | ~5 years | 20-59 y | Male Female | **ε4:** 67  **Total:** 294 | Cross- sectional | Self-report questionnaire (engagement in leisure time PA) | TC, HDL | The *APOE* ε4 allele associated with a high-risk lipid profile among males only. | Among females only, PA independently associated with a low-risk lipid profile. |
| Corella  (2001)^67^ | N/A | 18-66 y | Male Female | **ε4:** 119  **Total:** 819 | Cross-sectional | Self-report questionnaire (h/week engaged in leisure time PA) | HDL-C, LDL-C, TG | Significant interactions between *APOE* status and PA on HDL-C levels in males only, with higher HDL levels among active ε4 carriers vs. sedentary ε4 carriers. | Among males only, PA modulates the effect of *APOE* status on plasma lipid concentrations. |
| de Bruijn (2013)^68^ | ~8.8 y | 61-97 y | Male  Female | **ε4+:** ~1203  **Total:** 4406 | Prospective cohort | Adapted Zutphen Physical Activity questionnaire | Self-reported PA, dementia, AD incidence | NS | Non-significant association between PA and dementia risk. |
| Gelber (2012)^69^ | ~ 25 y | 71-93 y | Male | **ε4+:** 627  **Total:** 3468 | Case-control | Interview (mid-life h/day spent in no activity, sedentary, slight, moderate, and heavy activity) | Overall dementia, AD, VaD | Low-risk profile associated with lower dementia OR for *APOE* ε4 non-carriers, but not for carriers. | No association between individual factors and AD risk.  Significant association between individual factors (excluding diet) with VaD risk. |
| Gustavsson (2012)^70^ | NS | <75 y | Male Female | **ε4:** 1762  **Total:** 6389 | Retrospective cohort | Self-report questionnaire (engagement in leisure-time PA) | LDL-C, CHD risk | No interaction between *APOE* status and PA on LDL-C levels. | Non-significant interaction between *APOE* status and PA on CHD risk. |
| Lee (2018)^71^ | 6 months | 56.74 (9.92)  *(APOE* ɛ3: 56.80±10.65  *APOE* ɛ4: 56.67±9.19) | Male  Female | **ε4+:** 15  **Total:** 28 | Longitudinal | Aerobic exercise and resistance training (5 days/week: 45 min. aerobic exercise 3x/week; resistance training 2x/week) | Stroke risk factors (BP, lipid profile, Hcy); cognitive function (MMSE) | SBP decreased in *APOE* ε4 carriers after PA. | TC, LDL, and TG levels decreased, and HDL increased after PA, with higher reductions of TC, LDL, and TG in carriers vs. non-carriers.  Post exercise training, VO_2max_ and cognition increased (no sig. change between genotypes). |
| Niti (2008)^72^ | 1-2 y | ≥ 55 y | Male  Female | **ε4:** 292  **Total:** 1635 | Prospective cohort | Self-report questionnaire (engagement in leisure time PA) | Cognitive function (MMSE) | Interaction of *APOE* status and PA level on risk of cognitive decline, with more pronounced effects among *APOE* ε4 carriers. | Increased leisure activity, especially productive activities, associated with lowered risk of cognitive decline. |
| Piccarducci (2019)^73^ | N/A | 20-70 y | Male  Female | **ε4+:** 16  **Total:** 42 | Cross-sectional | Habits questionnaire | Oxidative status, Aβ levels, erythrocyte membrane composition | Active subjects displayed higher AOC than non-active subjects for *APOE* ε4 carriers only. | AOC, PC, and erythrocyte membrane fluidity were observed to be much lower in *APOE* ε4 carriers and non-active participants. The positive effects of PA on these biomarkers were more pronounced in non-ε4 carriers compared to *APOE* ε4 carriers. |
| Pisciotta (2003)^74^ | N/A | > 40 y  (Sedentary: 50. 8±7.2, Active:50.9+7 0) | Male | **ε4:** 27  **Total:** 200 | Cross- sectional | Aerobic physical activity (cycling 120-150 km/week) | HDL-C, LDL-C, TC, tHcy | In *APOE* ε4 carriers, active carriers had lower LDL-C levels compared to sedentary carriers. | Active males had lower LDL-C, TG, tHcy, increased HDL levels, and larger LDL particles compared to sedentary males. |
| Schmitz (2001)^75^ | ~7 y | 18-30 y | Male Female | **ε4:** ~1000  **Total:** 3626 | Prospective cohort | Graded Treadmill Test (CRF)  CARDIA Physical Activity History Questionnaire | HDL-C, LDL-C | No interaction observed between PA level and *APOE* status on plasma lipid levels. | Combination of independent effects of several variables (body weight, fitness, Key’s score, oral contraceptive use, alcohol intake, education, smoking) contribute to variance in lipid changes compared to that of *APOE* status. |
| Schuit (2001)^76^ | ~ 3 y | 65–84 y  74.6 + 4.3 | Male | **ε4+:** 79  **Total:** 347 | Prospective cohort | Self-report questionnaire (categorized as maximal 1 h/day vs. >1 h/day) | Cognitive function (MMSE) | Association between inactive *APOE* ε4 carriers and elevated risk of cognitive decline vs. active non-carriers. | Non-significant effect of PA on risk of cognitive decline. |
| Seip (2011)^77^ | 6 months | 18-70 y  (Men: 35.3 ± 10.7, Women: 38.2 ± 11.3 | Male  Female | **ε4+:** 54  **Total:** 166 | Prospective cohort | Aerobic exercise MTV training (40 min., 4x/week) | Lipase activities (HLA, LPLA) | PA training lowered HLA across all *APOE* genotypes.  LPLA levels post-exercise increased in the ε2/ε3 and ε4/ε3 groups, whereas levels decreased in the ε3/ε3 group (sig. only after correction for baseline insulin levels). | Interaction of *APOE* genotype with aerobic training to modulate LPLA irrespective of sex. |
| St-Amand (1999)^78^ | N/A | 25-48 y | Male Female | **ε4:** 29  **Total:** 129 | Cross-sectional | Cardiorespiratory fitness (V̇O_2max_) | HDL-C, LDL-C,  TC | No association between fitness level and lipoprotein-lipid levels in *APOE* ε4 carriers. | Higher physical fitness associated with favorable lipid profiles in non-ε4 carriers, with lower TG in ε2 carriers and higher HDL-C in ε3 homozygotes. |

**REFERENCES**

1.	Abbott RD, White LR, Ross GW, Masaki KH, Curb JD, Petrovitch H. Walking and dementia in physically capable elderly men. *Jama.* 2004;292(12):1447-1453.

2.	Boer JM, Ehnholm C, Menzel HJ, et al. Interactions between lifestyle-related factors and the ApoE polymorphism on plasma lipids and apolipoproteins. The EARS Study. European Atherosclerosis Research Study. *Arterioscler Thromb Vasc Biol.* 1997;17(9):1675-1681.

3.	Boots EA, Schultz SA, Oh JM, et al. Cardiorespiratory fitness is associated with brain structure, cognition, and mood in a middle-aged cohort at risk for Alzheimer's disease. *Brain Imaging Behav.* 2015;9(3):639-649.

4.	Brown BM, Peiffer JJ, Taddei K, et al. Physical activity and amyloid-β plasma and brain levels: results from the Australian Imaging, Biomarkers and Lifestyle Study of Ageing. *Mol Psychiatry.* 2013;18(8):875-881.

5.	Buchman AS, Boyle PA, Yu L, Shah RC, Wilson RS, Bennett DA. Total daily physical activity and the risk of AD and cognitive decline in older adults. *Neurology.* 2012;78(17):1323-1329.

6.	Chang M, Jonsson PV, Snaedal J, et al. The effect of midlife physical activity on cognitive function among older adults: AGES--Reykjavik Study. *J Gerontol A Biol Sci Med Sci.* 2010;65(12):1369-1374.

7.	Corlier F, Hafzalla G, Faskowitz J, et al. Systemic inflammation as a predictor of brain aging: Contributions of physical activity, metabolic risk, and genetic risk. *Neuroimage.* 2018;172:118-129.

8.	de Frutos-Lucas J, López-Sanz D, Zuluaga P, et al. Physical activity effects on the individual alpha peak frequency of older adults with and without genetic risk factors for Alzheimer's Disease: A MEG study. *Clin Neurophysiol.* 2018;129(9):1981-1989.

9.	de Frutos-Lucas J, Cuesta P, López-Sanz D, et al. The relationship between physical activity, apolipoprotein E ε4 carriage, and brain health. *Alzheimers Res Ther.* 2020;12(1):48.

10.	de Frutos-Lucas J, Cuesta P, Ramírez-Toraño F, et al. Age and APOE genotype affect the relationship between objectively measured physical activity and power in the alpha band, a marker of brain disease. *Alzheimers Res Ther.* 2020;12(1):113.

11.	Deeny SP, Poeppel D, Zimmerman JB, et al. Exercise, APOE, and working memory: MEG and behavioral evidence for benefit of exercise in epsilon4 carriers. *Biol Psychol.* 2008;78(2):179-187.

12.	Deeny SP, Winchester J, Nichol K, et al. Cardiovascular fitness is associated with altered cortical glucose metabolism during working memory in ɛ4 carriers. *Alzheimers Dement.* 2012;8(4):352-356.

13.	Etnier JL, Caselli RJ, Reiman EM, et al. Cognitive performance in older women relative to ApoE-epsilon4 genotype and aerobic fitness. *Med Sci Sports Exerc.* 2007;39(1):199-207.

14.	Fenesi B, Fang H, Kovacevic A, Oremus M, Raina P, Heisz JJ. Physical Exercise Moderates the Relationship of Apolipoprotein E (APOE) Genotype and Dementia Risk: A Population-Based Study. *J Alzheimers Dis.* 2017;56(1):297-303.

15.	Ferrari C, Xu WL, Wang HX, et al. How can elderly apolipoprotein E ε4 carriers remain free from dementia? *Neurobiol Aging.* 2013;34(1):13-21.

16.	Hansson O, Svensson M, Gustavsson AM, et al. Midlife physical activity is associated with lower incidence of vascular dementia but not Alzheimer's disease. *Alzheimers Res Ther.* 2019;11(1):87.

17.	Head D, Bugg JM, Goate AM, et al. Exercise Engagement as a Moderator of the Effects of APOE Genotype on Amyloid Deposition. *Arch Neurol.* 2012;69(5):636-643.

18.	Krell-Roesch J, Pink A, Roberts RO, et al. Timing of Physical Activity, Apolipoprotein E ε4 Genotype, and Risk of Incident Mild Cognitive Impairment. *J Am Geriatr Soc.* 2016;64(12):2479-2486.

19.	Kulmala J, Solomon A, Kåreholt I, et al. Association between mid- to late life physical fitness and dementia: evidence from the CAIDE study. *J Intern Med.* 2014;276(3):296-307.

20.	Larson EB, Wang L, Bowen JD, et al. Exercise is associated with reduced risk for incident dementia among persons 65 years of age and older. *Ann Intern Med.* 2006;144(2):73-81.

21.	Liang KY, Mintun MA, Fagan AM, et al. Exercise and Alzheimer's disease biomarkers in cognitively normal older adults. *Ann Neurol.* 2010;68(3):311-318.

22.	Lindsay J, Laurin D, Verreault R, et al. Risk factors for Alzheimer's disease: a prospective analysis from the Canadian Study of Health and Aging. *Am J Epidemiol.* 2002;156(5):445-453.

23.	Luck T, Riedel-Heller SG, Luppa M, et al. Apolipoprotein E epsilon 4 genotype and a physically active lifestyle in late life: analysis of gene-environment interaction for the risk of dementia and Alzheimer's disease dementia. *Psychol Med.* 2014;44(6):1319-1329.

24.	Paillard-Borg S, Fratiglioni L, Winblad B, Wang HX. Leisure activities in late life in relation to dementia risk: principal component analysis. *Dement Geriatr Cogn Disord.* 2009;28(2):136-144.

25.	Podewils LJ, Guallar E, Kuller LH, et al. Physical activity, APOE genotype, and dementia risk: findings from the Cardiovascular Health Cognition Study. *Am J Epidemiol.* 2005;161(7):639-651.

26.	Ravaglia G, Forti P, Lucicesare A, et al. Physical activity and dementia risk in the elderly: findings from a prospective Italian study. *Neurology.* 2008;70(19 Pt 2):1786-1794.

27.	Rovio S, Kåreholt I, Helkala EL, et al. Leisure-time physical activity at midlife and the risk of dementia and Alzheimer's disease. *Lancet Neurol.* 2005;4(11):705-711.

28.	Rovio S, Kåreholt I, Viitanen M, et al. Work-related physical activity and the risk of dementia and Alzheimer's disease. *Int J Geriatr Psychiatry.* 2007;22(9):874-882.

29.	Scarmeas N, Luchsinger JA, Schupf N, et al. Physical activity, diet, and risk of Alzheimer disease. *Jama.* 2009;302(6):627-637.

30.	Scarmeas N, Luchsinger JA, Brickman AM, et al. Physical activity and Alzheimer disease course. *Am J Geriatr Psychiatry.* 2011;19(5):471-481.

31.	Shaaban CE, Jia Y, Chang CH, Ganguli M. Independent and joint effects of vascular and cardiometabolic risk factor pairs for risk of all-cause dementia: a prospective population-based study. *Int Psychogeriatr.* 2019;31(10):1421-1432.

32.	Shih IF, Paul K, Haan M, Yu Y, Ritz B. Physical activity modifies the influence of apolipoprotein E ε4 allele and type 2 diabetes on dementia and cognitive impairment among older Mexican Americans. *Alzheimers Dement.* 2018;14(1):1-9.

33.	Smith JC, Nielson KA, Woodard JL, et al. Interactive effects of physical activity and APOE-ε4 on BOLD semantic memory activation in healthy elders. *Neuroimage.* 2011;54(1):635-644.

34.	Smith JC, Nielson KA, Woodard JL, et al. Physical activity reduces hippocampal atrophy in elders at genetic risk for Alzheimer's disease. *Front Aging Neurosci.* 2014;6:61.

35.	Smith JC, Lancaster MA, Nielson KA, et al. Interactive effects of physical activity and APOE-ε4 on white matter tract diffusivity in healthy elders. *Neuroimage.* 2016;131:102-112.

36.	Stojanovic M, Jin Y, Fagan AM, et al. Physical Exercise and Longitudinal Trajectories in Alzheimer Disease Biomarkers and Cognitive Functioning. *Alzheimer Dis Assoc Disord.* 2020;34(3):212-219.

37.	Taaffe DR, Irie F, Masaki KH, et al. Physical activity, physical function, and incident dementia in elderly men: the Honolulu-Asia Aging Study. *J Gerontol A Biol Sci Med Sci.* 2008;63(5):529-535.

38.	Tan ZS, Spartano NL, Beiser AS, et al. Physical Activity, Brain Volume, and Dementia Risk: The Framingham Study. *J Gerontol A Biol Sci Med Sci.* 2017;72(6):789-795.

39.	Tsai CL, Sun HS, Kuo YM, Pai MC. The Role of Physical Fitness in Cognitive-Related Biomarkers in Persons at Genetic Risk of Familial Alzheimer's Disease. *J Clin Med.* 2019;8(10).

40.	Tsai CL, Erickson KI, Sun HS, Kuo YM, Pai MC. A cross-sectional examination of a family history of Alzheimer's disease and ApoE epsilon 4 on physical fitness, molecular biomarkers, and neurocognitive performance. *Physiol Behav.* 2021;230:113268.

41.	Wilson RS, Bennett DA, Bienias JL, et al. Cognitive activity and incident AD in a population-based sample of older persons. *Neurology.* 2002;59(12):1910-1914.

42.	Woodard JL, Sugarman MA, Nielson KA, et al. Lifestyle and genetic contributions to cognitive decline and hippocampal structure and function in healthy aging. *Curr Alzheimer Res.* 2012;9(4):436-446.

43.	Zlatar ZZ, Wierenga CE, Bangen KJ, Liu TT, Jak AJ. Increased hippocampal blood flow in sedentary older adults at genetic risk for Alzheimer's disease. *J Alzheimers Dis.* 2014;41(3):809-817.

44.	Allard JS, Ntekim O, Johnson SP, et al. APOEε4 impacts up-regulation of brain-derived neurotrophic factor after a six-month stretch and aerobic exercise intervention in mild cognitively impaired elderly African Americans: A pilot study. *Exp Gerontol.* 2017;87(Pt A):129-136.

45.	Cheng ST, Chow PK, Song YQ, et al. Mental and physical activities delay cognitive decline in older persons with dementia. *Am J Geriatr Psychiatry.* 2014;22(1):63-74.

46.	Eggermont LH, Swaab DF, Hol EM, Scherder EJ. Walking the line: a randomised trial on the effects of a short term walking programme on cognition in dementia. *J Neurol Neurosurg Psychiatry.* 2009;80(7):802-804.

47.	Steen Jensen C, Portelius E, Siersma V, et al. Cerebrospinal Fluid Amyloid Beta and Tau Concentrations Are Not Modulated by 16 Weeks of Moderate- to High-Intensity Physical Exercise in Patients with Alzheimer Disease. *Dement Geriatr Cogn Disord.* 2016;42(3-4):146-158.

48.	Jensen CS, Bahl JM, Østergaard LB, et al. Exercise as a potential modulator of inflammation in patients with Alzheimer's disease measured in cerebrospinal fluid and plasma. *Exp Gerontol.* 2019;121:91-98.

49.	Karssemeijer EGA, Aaronson JA, Bossers WJR, Donders R, Olde Rikkert MGM, Kessels RPC. The quest for synergy between physical exercise and cognitive stimulation via exergaming in people with dementia: a randomized controlled trial. *Alzheimers Res Ther.* 2019;11(1):3.

50.	Sanders LMJ, Hortobágyi T, Karssemeijer EGA, Van der Zee EA, Scherder EJA, van Heuvelen MJG. Effects of low- and high-intensity physical exercise on physical and cognitive function in older persons with dementia: a randomized controlled trial. *Alzheimers Res Ther.* 2020;12(1):28.

51.	Carlson MC, Helms MJ, Steffens DC, Burke JR, Potter GG, Plassman BL. Midlife activity predicts risk of dementia in older male twin pairs. *Alzheimers Dement.* 2008;4(5):324-331.

52.	Gu Y, Beato JM, Amarante E, et al. Assessment of Leisure Time Physical Activity and Brain Health in a Multiethnic Cohort of Older Adults. *JAMA Netw Open.* 2020;3(11):e2026506.

53.	de Souto Barreto P, Andrieu S, Payoux P, Demougeot L, Rolland Y, Vellas B. Physical Activity and Amyloid-β Brain Levels in Elderly Adults with Intact Cognition and Mild Cognitive Impairment. *J Am Geriatr Soc.* 2015;63(8):1634-1639.

54.	Jeon SY, Byun MS, Yi D, et al. Midlife Lifestyle Activities Moderate APOE ε4 Effect on in vivo Alzheimer's Disease Pathologies. *Front Aging Neurosci.* 2020;12:42.

55.	Kerestes R, Phal PM, Steward C, et al. Alterations in dorsal and ventral posterior cingulate connectivity in APOE ε4 carriers at risk of Alzheimer's disease. *BJPsych Open.* 2015;1(2):139-148.

56.	Stringa N, van Schoor NM, Milaneschi Y, et al. Physical Activity as Moderator of the Association Between APOE and Cognitive Decline in Older Adults: Results from Three Longitudinal Cohort Studies. *J Gerontol A Biol Sci Med Sci.* 2020;75(10):1880-1886.

57.	Vemuri P, Lesnick TG, Przybelski SA, et al. Effect of intellectual enrichment on AD biomarker trajectories: Longitudinal imaging study. *Neurology.* 2016;86(12):1128-1135.

58.	Wang L, Larson EB, Bowen JD, van Belle G. Performance-based physical function and future dementia in older people. *Arch Intern Med.* 2006;166(10):1115-1120.

59.	Braskie MN, Boyle CP, Rajagopalan P, et al. Physical activity, inflammation, and volume of the aging brain. *Neuroscience.* 2014;273:199-209.

60.	Honea RA, Thomas GP, Harsha A, et al. Cardiorespiratory fitness and preserved medial temporal lobe volume in Alzheimer disease. *Alzheimer Dis Assoc Disord.* 2009;23(3):188-197.

61.	Yang SY, Weng PH, Chen JH, et al. Leisure activities, apolipoprotein E e4 status, and the risk of dementia. *J Formos Med Assoc.* 2015;114(12):1216-1224.

62.	Kivipelto M, Rovio S, Ngandu T, et al. Apolipoprotein E epsilon4 magnifies lifestyle risks for dementia: a population-based study. *J Cell Mol Med.* 2008;12(6b):2762-2771.

63.	Tolppanen AM, Solomon A, Kulmala J, et al. Leisure-time physical activity from mid- to late life, body mass index, and risk of dementia. *Alzheimers Dement.* 2015;11(4):434-443.e436.

64.	Espeland MA, Luchsinger JA, Baker LD, et al. Effect of a long-term intensive lifestyle intervention on prevalence of cognitive impairment. *Neurology.* 2017;88(21):2026-2035.

65.	Bernstein MS, Costanza MC, James RW, et al. Physical activity may modulate effects of ApoE genotype on lipid profile. *Arterioscler Thromb Vasc Biol.* 2002;22(1):133-140.

66.	Boer JM, Feskens EJ, Schouten EG, Havekes LM, Seidell JC, Kromhout D. Lipid profiles reflecting high and low risk for coronary heart disease: contribution of apolipoprotein E polymorphism and lifestyle. *Atherosclerosis.* 1998;136(2):395-402.

67.	Corella D, Guillén M, Sáiz C, et al. Environmental factors modulate the effect of the APOE genetic polymorphism on plasma lipid concentrations: ecogenetic studies in a Mediterranean Spanish population. *Metabolism.* 2001;50(8):936-944.

68.	de Bruijn RF, Schrijvers EM, de Groot KA, et al. The association between physical activity and dementia in an elderly population: the Rotterdam Study. *Eur J Epidemiol.* 2013;28(3):277-283.

69.	Gelber RP, Petrovitch H, Masaki KH, et al. Lifestyle and the risk of dementia in Japanese-american men. *J Am Geriatr Soc.* 2012;60(1):118-123.

70.	Gustavsson J, Mehlig K, Leander K, et al. Interaction of apolipoprotein E genotype with smoking and physical inactivity on coronary heart disease risk in men and women. *Atherosclerosis.* 2012;220(2):486-492.

71.	Lee JH, Hong SM, Shin YA. Effects of exercise training on stroke risk factors, homocysteine concentration, and cognitive function according the APOE genotype in stroke patients. *J Exerc Rehabil.* 2018;14(2):267-274.

72.	Niti M, Yap KB, Kua EH, Tan CH, Ng TP. Physical, social and productive leisure activities, cognitive decline and interaction with APOE-epsilon 4 genotype in Chinese older adults. *Int Psychogeriatr.* 2008;20(2):237-251.

73.	Piccarducci R, Daniele S, Fusi J, et al. Impact of ApoE Polymorphism and Physical Activity on Plasma Antioxidant Capability and Erythrocyte Membranes. *Antioxidants (Basel).* 2019;8(11).

74.	Pisciotta L, Cantafora A, Piana A, et al. Physical activity modulates effects of some genetic polymorphisms affecting cardiovascular risk in men aged over 40 years. *Nutr Metab Cardiovasc Dis.* 2003;13(4):202-210.

75.	Schmitz KH, Schreiner PJ, Jacobs DR, et al. Independent and interactive effects of apolipoprotein E phenotype and cardiorespiratory fitness on plasma lipids. *Ann Epidemiol.* 2001;11(2):94-103.

76.	Schuit AJ, Feskens EJ, Launer LJ, Kromhout D. Physical activity and cognitive decline, the role of the apolipoprotein e4 allele. *Med Sci Sports Exerc.* 2001;33(5):772-777.

77.	Seip RL, Zoeller RF, Angelopoulos TJ, et al. Interactive effects of APOE haplotype, sex, and exercise on postheparin plasma lipase activities. *J Appl Physiol (1985).* 2011;110(4):1021-1028.

78.	St-Amand J, Prud'homme D, Moorjani S, et al. Apolipoprotein E polymorphism and the relationships of physical fitness to plasma lipoprotein-lipid levels in men and women. *Med Sci Sports Exerc.* 1999;31(5):692-697.
