# Supplementary material for: APOE ε4, physical activity, and the brain: a review of systematic reviews
Source: Front Aging Neurosci. 2026 Jul 23;18:1798639. doi: 10.3389/fnagi.2026.1798639 (PMC13442396; doi:10.3389/fnagi.2026.1798639)
Supplement: Supplementary file 2 [file Data_Sheet_2.DOCX]

| **1.** | **PubMed/Medline** | ("dementia"[MeSH Terms] OR "cognitive aging"[MeSH Terms] OR “neurodegenerative disorder”[Title/Abstract] OR "dementia"[Title/Abstract] OR "dementias"[Title/Abstract] OR "amentia"[Title/Abstract] OR "amentias"[Title/Abstract] OR "Alzheimer*"[Title/Abstract] OR "cognitive decline"[Title/Abstract] OR "cognitive impairment"[Title/Abstract] OR "cognitive aging"[Title/Abstract] OR "cognitive ageing"[Title/Abstract] OR "cognitive declines"[Title/Abstract] OR "cognitive impairments"[Title/Abstract] OR "cognitive dysfunction"[Title/Abstract] OR "cognitive dysfunctions"[Title/Abstract] OR "cognitive disorder"[Title/Abstract] OR "cognitive disorders"[Title/Abstract] OR "mental deterioration"[Title/Abstract] OR “brain derived neurotrophic factor”[Title/Abstract] OR “neurodegeneration”[Title/Abstract] OR “AD risk factor”[Title/Abstract] OR “beta-amyloid plaque”[Title/Abstract] OR “beta-amyloid plaques”[Title/Abstract])  AND  (“exercise”[MeSH Terms] OR “aerobic exercise”[MeSH Terms] OR “anaerobic exercise”[MeSH Terms] OR “circuit-based exercise”[MeSH Terms] OR “exercise therapy”[MeSH Terms] OR “cool-down exercise”[MeSH Terms] OR “warm-up exercise”[MeSH Terms] OR “plyometric exercise”[MeSH Terms] OR “exercise movement techniques”[MeSH Terms] OR “cardiorespiratory fitness”[MeSH Terms] OR “physical fitness”[MeSH Terms] OR “isometric exercise”[MeSH Terms] OR “physical exercise”[MeSH Terms] OR “exercise training”[MeSH Terms] OR “physical activity”[MeSH Terms] OR “exercise”[Title/Abstract] OR “aerobic exercise”[Title/Abstract] OR “anaerobic exercise”[Title/Abstract] OR “circuit-based exercise”[Title/Abstract] OR “exercise therapy”[Title/Abstract] OR “cool-down exercise”[Title/Abstract] OR “warm-up exercise”[Title/Abstract] OR “plyometric exercise”[Title/Abstract] OR “exercise movement techniques”[Title/Abstract] OR “cardiorespiratory fitness”[Title/Abstract] OR “physical fitness”[Title/Abstract] OR “isometric exercise”[Title/Abstract] OR “physical exercise”[Title/Abstract] OR “exercise training”[Title/Abstract] OR “physical activity”[Title/Abstract] OR “strength training”[Title/Abstract] OR “exercise-induced hormones”[Title/Abstract])  Fitness  Resistance  VO2 Max  AND  (“apolipoprotein e4”[MeSH Terms] OR “apolipoprotein e4”[Title/Abstract] OR “APOE4”[Title/Abstract] OR “APO Epsilon 4”[Title/Abstract] OR “APO E-4”[Title/Abstract] OR “APO E 4”[Title/Abstract] OR “apolipoprotein e4”[Title/Abstract] OR “apolipoprotein epsilon4”[Title/Abstract] OR “apoe epsilon4”[Title/Abstract])  AND  (Meta-Analysis [Publication Type] OR systematic review[Filter] OR "systematic review" OR "systematic reviews" OR “meta-analysis” OR “metaanalysis” OR “metanalysis” OR “meta-analyses” OR “metaanalyses” OR “metanalyses” OR “PRISMA”) |
| --- | --- | --- |
| **2.** | **Embase/Elsevier** | (‘apolipoprotein e4’/exp OR ‘apolipoprotein e4’:ti,ab OR ‘APOE4’:ti,ab OR ‘APO Epsilon 4’:ti,ab OR ‘APO E-4’:ti,ab OR ‘APO E 4’:ti,ab OR ‘apolipoprotein e4’:ti,ab OR ‘apolipoprotein epsilon4’:ti,ab OR ‘apoe epsilon4’:ti,ab)  AND  (‘dementia’/exp OR ‘cognitive aging’/exp OR ‘neurodegenerative disorder’:ti,ab OR ‘dementia’:ti,ab OR ‘dementias’:ti,ab OR ‘amentia’:ti,ab OR ‘amentias’:ti,ab OR ‘Alzheimer*’:ti,ab OR ‘cognitive decline’:ti,ab OR ‘cognitive impairment’:ti,ab OR ‘cognitive aging’:ti,ab OR ‘cognitive ageing’:ti,ab OR ‘cognitive declines’:ti,ab OR ‘cognitive impairments’:ti,ab OR ‘cognitive dysfunction’:ti,ab OR ‘cognitive dysfunctions’:ti,ab OR ‘cognitive disorder’:ti,ab OR ‘cognitive disorders’:ti,ab OR ‘mental deterioration’:ti,ab OR ‘brain derived neurotrophic factor’:ti,ab OR ‘neurodegeneration’:ti,ab OR ‘AD risk factor’:ti,ab OR ‘beta-amyloid plaque’:ti,ab OR ‘beta-amyloid plaques’:ti,ab)  AND  (‘exercise’/exp OR ‘aerobic exercise’/exp OR ‘anaerobic exercise’/exp OR ‘circuit-based exercise’/exp OR ‘exercise therapy’/exp OR ‘cool-down exercise’/exp OR ‘warm-up exercise’/exp OR ‘plyometric exercise’/exp OR ‘exercise movement techniques’/exp OR ‘cardiorespiratory fitness’/exp OR ‘physical fitness’/exp OR ‘isometric exercise’/exp OR ‘physical exercise’/exp OR ‘exercise training’/exp OR ‘physical activity’/exp OR ‘exercise’:ti,ab OR ‘aerobic exercise’:ti,ab OR ‘anaerobic exercise’:ti,ab OR ‘circuit-based exercise’:ti,ab OR ‘exercise therapy’:ti,ab OR ‘cool-down exercise’:ti,ab OR ‘warm-up exercise’:ti,ab OR ‘plyometric exercise’:ti,ab OR ‘exercise movement techniques’:ti,ab OR ‘cardiorespiratory fitness’:ti,ab OR ‘physical fitness’:ti,ab OR ‘isometric exercise’:ti,ab OR ‘physical exercise’:ti,ab OR ‘exercise training’:ti,ab OR ‘exercise training’:ti,ab OR ‘physical activity’:ti,ab OR ‘strength training’:ti,ab OR ‘exercise-induced hormones’:ti,ab)  AND  ('meta analysis'/exp OR 'systematic review'/exp OR 'systematic review':ti,ab OR 'systematic reviews':ti,ab OR 'meta analysis':ti,ab OR 'metaanalysis':ti,ab OR 'metanalysis':ti,ab OR 'meta analyses':ti,ab OR 'metaanalyses':ti,ab OR 'metanalyses':ti,ab OR PRISMA:ti,ab)(‘apolipoprotein e4’/exp OR ‘apolipoprotein e4’:ti,ab OR ‘APOE4’:ti,ab OR ‘APO Epsilon 4’:ti,ab OR ‘APO E-4’:ti,ab OR ‘APO E 4’:ti,ab OR ‘apolipoprotein e4’:ti,ab OR ‘apolipoprotein epsilon4’:ti,ab OR ‘apoe epsilon4’:ti,ab) AND (‘dementia’/exp OR ‘cognitive aging’/exp OR ‘neurodegenerative disorder’:ti,ab OR ‘dementia’:ti,ab OR ‘dementias’:ti,ab OR ‘amentia’:ti,ab OR ‘amentias’:ti,ab OR ‘Alzheimer*’:ti,ab OR ‘cognitive decline’:ti,ab OR ‘cognitive impairment’:ti,ab OR ‘cognitive aging’:ti,ab OR ‘cognitive ageing’:ti,ab OR ‘cognitive declines’:ti,ab OR ‘cognitive impairments’:ti,ab OR ‘cognitive dysfunction’:ti,ab OR ‘cognitive dysfunctions’:ti,ab OR ‘cognitive disorder’:ti,ab OR ‘cognitive disorders’:ti,ab OR ‘mental deterioration’:ti,ab OR ‘brain derived neurotrophic factor’:ti,ab OR ‘neurodegeneration’:ti,ab OR ‘AD risk factor’:ti,ab OR ‘beta-amyloid plaque’:ti,ab OR ‘beta-amyloid plaques’:ti,ab) AND (‘exercise’/exp OR ‘aerobic exercise’/exp OR ‘anaerobic exercise’/exp OR ‘circuit-based exercise’/exp OR ‘exercise therapy’/exp OR ‘cool-down exercise’/exp OR ‘warm-up exercise’/exp OR ‘plyometric exercise’/exp OR ‘exercise movement techniques’/exp OR ‘cardiorespiratory fitness’/exp OR ‘physical fitness’/exp OR ‘isometric exercise’/exp OR ‘physical exercise’/exp OR ‘exercise training’/exp OR ‘physical activity’/exp OR ‘exercise’:ti,ab OR ‘aerobic exercise’:ti,ab OR ‘anaerobic exercise’:ti,ab OR ‘circuit-based exercise’:ti,ab OR ‘exercise therapy’:ti,ab OR ‘cool-down exercise’:ti,ab OR ‘warm-up exercise’:ti,ab OR ‘plyometric exercise’:ti,ab OR ‘exercise movement techniques’:ti,ab OR ‘cardiorespiratory fitness’:ti,ab OR ‘physical fitness’:ti,ab OR ‘isometric exercise’:ti,ab OR ‘physical exercise’:ti,ab OR ‘exercise training’:ti,ab OR ‘physical activity’:ti,ab OR ‘strength training’:ti,ab OR ‘exercise-induced hormones’:ti,ab) AND ('meta analysis'/exp OR 'systematic review'/exp OR 'systematic review':ti,ab OR 'systematic reviews':ti,ab OR 'meta analysis':ti,ab OR 'metaanalysis':ti,ab OR 'metanalysis':ti,ab OR 'meta analyses':ti,ab OR 'metaanalyses':ti,ab OR 'metanalyses':ti,ab OR PRISMA:ti,ab) |
| **3.** | **Web of Science** | (“APOE4” OR “APO E4” OR “APO Epsilon 4” OR “APO E-4” OR “APO E 4” OR “apolipoprotein epsilon4” OR “apolipoprotein e4” OR “apoe epsilon4”)  AND  (“neurodegenerative disorder” OR “dementia” OR “dementias” OR “amentia” OR “amentias” OR “Alzheimer*” OR “cognitive decline” OR “cognitive impairment” OR “cognitive aging” OR “cognitive ageing” OR “cognitive declines” OR “cognitive impairments” OR “cognitive disorders” OR “mental deterioration” OR “brain derived neurotrophic factor” OR “neurodegeneration” OR “AD risk factor” OR “beta-amyloid plaque” OR “beta-amyloid plaques”)  AND  (“exercise*” OR “aerobic exercise” OR “anaerobic exercise” OR “circuit-based exercise” OR “exercise therapy” OR “cool-down exercise” OR “warm-up exercise” OR “plyometric exercise” OR “exercise movement techniques” OR “cardiorespiratory fitness” OR “physical fitness” OR “isometric exercise” OR “physical exercise” OR “exercise training” OR “physical activity” OR “strength training” OR “exercise-induced hormones”)  AND  (“systematic review” OR “systematic reviews” OR “meta analysis” OR “metaanalysis” OR “metanalysis” OR “meta analyses” OR “metaanalyses” OR “metanalyses” OR PRISMA) |
| **4.** | **Scopus/Elsevier** | (“APOE4” OR “APO E4” OR “APO Epsilon 4” OR “APO E-4” OR “APO E 4” OR “apolipoprotein epsilon4” OR “apolipoprotein e4” OR “apoe epsilon4”) AND (“neurodegenerative disorder” OR “dementia” OR “dementias” OR “amentia” OR “amentias” OR “Alzheimer*” OR “cognitive decline” OR “cognitive impairment” OR “cognitive aging” OR “cognitive ageing” OR “cognitive declines” OR “cognitive impairments” OR “cognitive disorders” OR “mental deterioration” OR “brain derived neurotrophic factor” OR “neurodegeneration” OR “AD risk factor” OR “beta-amyloid plaque” OR “beta-amyloid plaques”) AND (“exercise*” OR “aerobic exercise” OR “anaerobic exercise” OR “circuit-based exercise” OR “exercise therapy” OR “cool-down exercise” OR “warm-up exercise” OR “plyometric exercise” OR “exercise movement techniques” OR “cardiorespiratory fitness” OR “physical fitness” OR “isometric exercise” OR “physical exercise” OR “exercise training” OR “physical activity” OR “strength training” OR “exercise-induced hormones”) AND (“systematic review” OR “systematic reviews” OR “meta analysis” OR “metaanalysis” OR “metanalysis” OR “meta analyses” OR “metaanalyses” OR “metanalyses” OR PRISMA) |
